# Crosstalk of peripheral cytokine-white matter alteration-insomnia during methadone maintenance treatment

**DOI:** 10.1017/S0033291726103614

**Published:** 2026-04-27

**Authors:** Longtao Yang, Wenhan Yang, Yihui Tang, Yule Zeng, Suiling Liu, Zhentao Gao, Xiaoying Li, Jun Liu

**Affiliations:** Department of Radiology, The Second Xiangya Hospital of Central South University, Changsha, Hunan, China

**Keywords:** diffusion tensor imaging, interferon-γ, insomnia, methadone maintenance treatment, opioid use disorder, white matter

## Abstract

**Background:**

Insomnia is commonly seen in opioid use disorder (OUD) patients receiving methadone maintenance treatment (MMT) and might be related to high heroin relapse risk. This study aims to identify potential mediation pathways among peripheral cytokines, neuroimaging characteristics, and insomnia in MMT patients, and explore diagnostic markers and therapeutic targets for MMT-related insomnia.

**Methods:**

A total of 121 OUD individuals (OUDs) and 109 healthy controls were recruited, including MMT individuals (MMT group, *N* = 53), short-term abstinent (median: 30 days) heroin users at baseline (OUD1, *N* = 68), and around 10-month follow-up (OUD2, *N* = 61) without MMT, healthy controls-cohort 1 (HC1, *N* = 53, age/gender/education match MMT), and healthy controls-cohort 2 (HC2, *N* = 56, age/gender match OUD1). Multimodal datasets, including cerebral diffusion tensor imaging (DTI), peripheral hematologic indicators, and neuropsychological assessments, were collected from the MMT group and HC1. Within the MMT group, we revealed relationships among cytokines, DTI metrics, and neuropsychological assessments via partial correlation and mediation analyses. Mendelian randomization (MR) analyses between OUD and white matter (WM)-related imaging-derived phenotypes were used to further confirm Tract-Based Spatial Statistics (TBSS) results. Besides, the results of TBSS among OUD1, OUD2, and HC2 hypothetically served as baseline WM alteration before MMT.

**Results:**

Through comparisons among OUD1, OUD2, and HC2, WM aberrances could return to normal after 10-month abstinence, and we used the results as baseline alterations before MMT. MMT patients exhibited a broad imbalance in peripheral immune cells and cytokines, as well as presented insomnia, anxiety, and depression symptoms. After Bonferroni correction, mean diffusivity and radial diffusivity in extensive WM regions were higher in MMT patients than those of HC1. Ultimately, through multimodal correlation analysis, the ‘Interferon-γ (IFN-γ)-WM aberrance-insomnia’ axis was discovered in the MMT group.

**Conclusions:**

Together, these results primarily link cytokines and WM injury for OUDs with MMT to insomnia, implicating pharmacological IFN-γ target as a latent strategy to improve the insomnia of MMT patients.

## Graphical abstract

**Figure:**
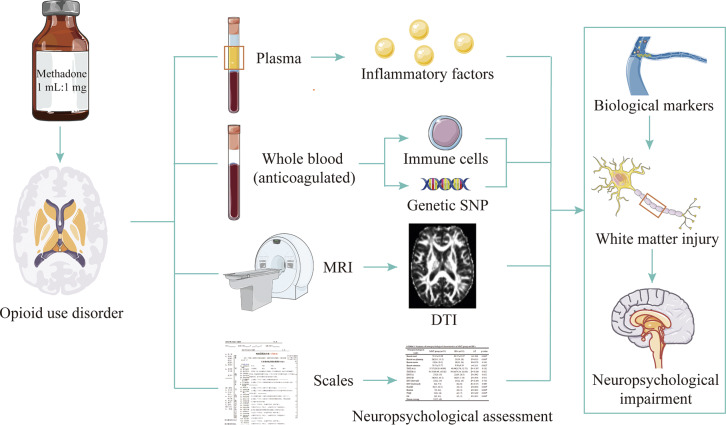

## Introduction

According to the World Drug Report 2024 released by the United Nations Office on Drugs and Crime, opioids are consumed by ~60 million individuals worldwide, with heroin accounting for nearly 50%. As an international crisis, opioid use disorder (OUD) is a treatable chronic encephalopathy characterized by cycles of relapse and remission, involving compulsive opioid use despite adverse consequences (Taylor & Samet, 2022). With the strongest evidence for effectiveness, methadone, a full opioid agonist, serves as a substituted treatment medicine for opioid use (Bell & Strang, 2020). During the methadone maintenance treatment (MMT) period, over 60% OUDs often exhibited neuropsychological symptoms (e.g., insomnia, depression, and anxiety), which were correlated with poor prognosis (Alikhani et al., 2020; Baldassarri et al., 2020; Dolsen & Harvey, 2017; Zankl, Martin, Davey, & Osborn, 2021). For example, sleep is crucial for recovery and rest across different biological functions, and its disturbance could elevate the risk of opioid relapse (Tripathi et al., 2020). However, the potential neuroimaging and peripheral immune alterations of MMT-related neuropsychological abnormality were still unclear, and further comprehension will facilitate life quality and relapse prevention of OUDs by fostering mental wellness.

As one of the neuroimaging techniques reflecting neuroinflammation, diffusion tensor imaging (DTI) enables the noninvasive, in vivo evaluation of microstructural white matter (WM) organization (Aronica et al., 2022). Specifically, fractional anisotropy (FA) represents the directional preference of water diffusion within a voxel, while mean diffusivity (MD) indexes the mean magnitude of water diffusion. Axial diffusivity (AD; L1) and radial diffusivity (RD) [(L2 + L3)/2], respectively, quantify water diffusion along and perpendicular to the principal axis of the diffusion tensor (Tohyama et al., 2025). Reduced FA indicates axonal myelin sheath degradation associated with oligodendrocytes (Molloy, Nugent, & Bokde, 2021), while elevated MD and RD related to various excitatory neurons reflect an increase in the averaged diffusion rate and a decrease in overall cell density (Shen et al., 2025). Decrease in WM integrity was positively related to accumulated methadone intake (Haghighi-Morad et al., 2020).

Notably, OUD not only alters patients’ neuroimaging characteristics but also exerts adverse immunomodulatory effects on the immune system (Plein & Rittner, 2018). Long-term MMT may have complex impacts on the peripheral immunity of OUD patients. Chronic methadone altered CD8+ T-cell phenotype in vivo and modulated its in vitro responses to opioid receptor and T-cell receptor stimulation; specifically, methadone use reduces the frequency of terminally differentiated effector memory CD8^+^ T cells, suggesting that methadone may affect the differentiation and activation status of CD8^+^ T cells (Mazahery, Benson, Cruz-Lebrón, & Levine, 2020). Among heroin-dependent individuals receiving MMT, CD4^+^ T-cell count is an important variable (Quang-Cantagrel, Wallace, Ashar, & Mathews, 2001; Wu et al., 2020). On the contrary, long-term methadone use is also linked to immune activation and elevated inflammatory levels: It has been reported that the MMT group exhibited higher plasma Interleukin-1β/6/8 (IL-1β/6/8) than healthy controls, where IL-6 and IL-1β were positively correlated with methadone daily dose and MMT duration, respectively (Chan et al., 2015). Although no further studies have yet explored the effects of MMT and opioid addiction on lymphocyte functional activation, the chronic inflammation induced by long-term MMT may disrupt the balance and functional activation of lymphocyte subsets through the imbalanced cytokine network. Therefore, more refined lymphocyte markers warrant further investigation. More importantly, few studies explored the association between peripheral blood immunoinflammatory levels and brain DTI features. Clarifying whether WM damage mediated the relationships between peripheral immunoinflammatory conditions and neuropsychological performance will provide latent biomarkers for diagnosis and targeted therapy of MMT-related neuropsychological symptoms. In addition, hereditary factors like genetic single-nucleotide polymorphisms (SNPs) (e.g., rs6902403 and rs933271) might mediate the susceptibility to MMT effects (Duan et al., 2020; Peng et al., 2018), thereby possibly impacting alterations in brain features. Besides, our previous work indicated a genetic basis between inflammatory cytokines and diffusivity-related imaging-derived phenotypes (IDPs) (Yang et al., 2026). Thus, associations of SNP phenotypes and vulnerability to WM damage during MMT needed to be taken into consideration.

The identification of diagnostic biomarkers and therapeutic targets associated with neuropsychological aberrances during MMT remains a critical priority in OUD treatment. Thus, this study focused on the combination of WM neuroimaging findings with multimodal clinical and biological datasets in OUDs with MMT, such as neuropsychological scales, peripheral blood immune indexes, and SNP genotypes. Abstinent heroin users at baseline (OUD1) and at 10-month follow-up (OUD2) were included as the baseline cohort before MMT. Moreover, Mendelian randomization (MR) analyses were further used to confirm associations between OUD and 384 WM-related IDPs, involving FA, MD, L1, L2, L3, intracellular volume fraction (ICVF), isotropic volume fraction (ISOVF), and orientation dispersion index (ODI). A multimodal integration approach shows promise for optimizing therapeutic protocols and enabling real-time monitoring of treatment responses via clarification of ‘biomarker-neuroimaging-neuropsychology’ axis, which will improve clinical outcomes by facilitating individual brain rehabilitation and thereby preventing relapse.

## Methods and materials

### Ethics approval and consent to participate

The Institutional Review Board of the Second Xiangya Hospital, Central South University, approved the protocol (number: 8167071216). Written informed consent was obtained from participants after the experimental procedure was explained and after they read the consent form.

### Study population

A total of 230 subjects were involved in this study, including the MMT group (*N* = 53), healthy controls-cohort 1 (HC1) (*N* = 53), OUD1 (*N* = 68, follow-up *N* = 61), and healthy controls-cohort 1 (HC2) (*N* = 56). MMT group who had returned to society were admitted to the detoxification center (Tianxin and Kangda in Changsha, Hunan, China) and received MMT without buprenorphine treatment. They even tested positive for heroin in urine screenings and met the diagnostic criteria for OUD as defined by the fifth version of the Diagnostic and Statistical Manual of Mental Disorders. In addition, the absence of ketamine and methamphetamine in urine tests was required as an inclusion criterion. Age-, gender-, body mass index- (BMI-), education-, Fagerstrom Test for Nicotine Dependence (FTND)-, and Alcohol Use Disorders Identification Test (AUDIT)-matched HC1 (*N* = 53) were also voluntarily recruited. All subjects were right-handed and had Han Chinese ancestry. No previous diagnoses of structural brain disease, head trauma, seizures, behavioral or mental disorders, or magnetic resonance imaging (MRI) contraindications were reported in both the MMT group and HC1. On the same day, each subject signed written consent forms and took part in the MRI scan, questionnaire, and peripheral blood acquisition.

**Figure 1.**
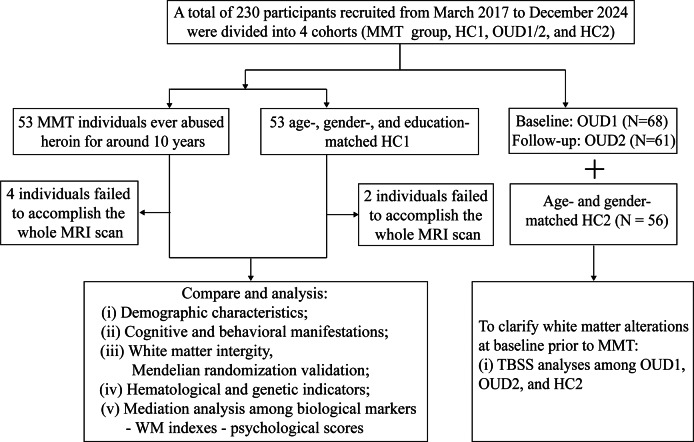
A workflow picture of this study. This work centered on white matter (WM)-related neuroimaging alterations of methadone maintenance treatment (MMT) individuals and combined with multimodal datasets, including neuropsychological and hematological (such as immune cell and inflammatory factor) data to discover relationships among biological markers-WM indexes-psychological scores. The DTI datasets of other cohorts, including OUD1, OUD2, and HC2, were used to investigate the longitudinal effects of abstinence (non-MMT intervention) on WM integrity. DTI, diffusion tensor imaging; HC1, healthy controls-cohort 1; HC2, healthy controls-cohort 2; OUD1, abstinent heroin users at baseline; OUD2, abstinent heroin users at around 10-month follow-up; MRI, magnetic resonance imaging; SNP, single-nucleotide polymorphism. Figure 1 is adapted from Servier Medical Art with publication permission.

Without overlapping subjects with the MMT group, OUD1 (Age [mean: 41.94 years] that was approximately similar to age of MMT initiation in the MMT group), with a history of heroin use, was recruited from drug rehabilitation clinics in Zhuzhou, PingTang, and Xinkaipu (three cities in Hunan Province, China) between March 2017 and December 2023 and was hypothetically taken as baseline situation of the MMT group. OUD1 were admitted to the detoxification center and received 10 months of education and physical training without MMT. The OUD2 group was a longitudinal follow-up cohort of OUD1. Age- and gender-matched HC2 were recruited without overlapping subjects with HC1. The general inclusion and exclusion criteria of OUD1 and HC2 were, respectively, the same as those of MMT and HC1. Only demographic and DTI information of OUD1, OUD2, and HC2 were provided in this study. An overview of the study flow chart is shown in Figure 1.

### Acquisition of MRI data

All above participants completed MRI scanning on a 3T MRI scanner (MAGNETOM Skyra, Siemens Healthcare, Erlangen, Germany) equipped with a 32-channel phased-array head coil at the Second Xiangya Hospital of Central South University, Changsha, China. During the scanning procedure, forehead restraining strap and foam padding were applied to stabilize participants’ heads for minimizing motion artifacts. They were also required to remain as motionless as possible in a supine position throughout the acquisition to ensure optimal image quality. Macroscopic brain abnormalities were assessed and excluded using fluid-attenuated inversion recovery sequences and T2-weighted imaging as part of the structural imaging protocol. DTI scans lasting 12 minutes and 13 seconds were collected along 64 directions (*b* = 1,000s/mm^2^), together with an acquisition without diffusion weighting (*b* = 0 s/mm^2^), using the following parameters: repetition time (TR) = 9500 ms, echo time (TE) = 88 ms, voxel size = 2 × 2 × 2 mm^3^, field of view (FOV) = 256 × 256 mm^2^, slice thickness = 2 mm, slices = 60, and slice spacing = 1 mm.

### Neuropsychological test acquisition

The MMT group and HC1 underwent these 15 neuropsychological tests on the same day as the MRI scan: (i–) Barratt impulsiveness scale-11, a 30-item self-report instrument designed to assess impulsiveness, is scored on a 4-point Likert scale from 1 (*rarely/never*) to 4 (*almost always*) and consists of three parts including Barratt-no-planning, Barratt-motor, and Barratt-attention. (v–viii) The Brief Cognitive Assessment Tool for Schizophrenia (B-CATS) represents an efficient neuropsychological battery that exhibits robust psychometric characteristics, showing a 0.76 correlation with the MATRICS Consensus Cognitive Battery, along with outstanding reliability across repeated administrations. The B-CATS includes Trail-Making Test Part A/B (TMT-A/B) and Digit Symbol Substitution Test (DSST), which are widely used to assess executive functions. The TMT incorporates two components: Part A and Part B. In Part A, it involves 25 numbered circles displayed in random order, requiring participants to draw lines connecting the numbers in increasing sequence (1-2-3-…). Part B elevates cognitive requirements by introducing a task where participants must sequentially connect numbers and letters in alternating ascending sequences (1-A-2-B-…). The experimental protocol requires participants to maintain continuous contact between the pen and paper during both task components. Task performance was measured by calculating the overall duration, incorporating both execution time and any error correction periods. The shorter time indicates better performance. The DSST measured participants’ ability to quickly match numbers to corresponding symbols during a 2-minute interval, with the final score reflecting the quantity of correctly identified pairs. Higher scores on the DSST reflect better cognitive performance. (ix and x) The Digit Span Test comprised two components: the forward span task, which measured attentional capacity, and the backward span task, which evaluated working memory function. (xi and xii) The Hamilton anxiety scale (HAMA, whose scores of 7 ∼ <20 points, suggesting anxiety susceptibility) and Hamilton depression scale (HAMD, whose scores of 8 ∼ < 20 points indicate depression predisposition) were reliably used to assess emotional status. Symptom severity was positively related to increasing scores. (xiii and xiv) The sleep quality is assessed via Pittsburgh Sleep Quality Index (PSQI) (Chinese version), which consists of 18 items across 7 domains. The overall score ranges from 0 to 21, with elevated scores reflecting poorer sleep quality. Insomnia severity is measured by seven-item Insomnia Severity Index (ISI), capturing participants’ subjective insomnia experiences, with a total score < 8 denoting normal sleep, 8 ~ 14 indicating subthreshold insomnia, 15 ~ 21 representing moderate insomnia, and 22 ~ 28 signifying severe insomnia. (xv) The intensity of current heroin urges and cravings is assessed using the Heroin Craving Questionnaire, where elevated scores correspond to stronger drug cravings (Dürsteler-MacFarland et al., 2012; Kleykamp et al., 2019; Tiffany, Carter, & Singleton, 2000).

### Flow cytometric analysis of immune cell phenotypes

To analyze immune cell populations in the blood of the MMT group and HC1, flow cytometry was employed using a series of human-specific antibodies. The antibodies used and their relevant details are as follows: Anti-HLA-DR (FITC-conjugated, Clone: L243), Anti-CD28 (PE-conjugated, Clone: CD28.2), Anti-CD4 (PerCP-conjugated, Clone: SK3), Anti-CD45RA (PE-CY7-conjugated, Clone: HI100), Anti-CD8 (APC-conjugated, Clone: RPA-T8), Anti-CD3 (APC-Cy7-conjugated, Clone: SK7), Anti-CD38 (BV421-conjugated, Clone: HIT2), Anti-CD45 (BV510-conjugated, Clone: HI30), Anti-CD45RA (FITC-conjugated, Clone: HI100), Anti-CD127 (PE-conjugated, Clone: hIL-7R-M21), Anti-CD25 (APC-conjugated, Clone: M-A251), Anti-IgD (FITC-conjugated, Clone: IA6–2), Anti-CD27 (PerCP-Cy5.5-conjugated, Clone: M-T271), Anti-CD19 (PE-Cy7-conjugated, Clone: SJ25C1), and Anti-CD20 (APC-conjugated, Clone: 2H7). All of these antibodies were purchased from BD Biosciences, Franklin Lakes, New Jersey, USA, and were used for the identification and analysis of immune cell subsets in the blood of participants. All samples were analyzed using the BD FACSCanto II flow cytometer (BD Biosciences, Franklin Lakes, New Jersey, USA) for quantitative detection.

Peripheral blood from the MMT group and HC1 was incubated with the relevant antibodies at room temperature and protected from light, for 30 minutes. After incubation, 1 mL of Phosphate Buffered Saline (PBS) (NCM biotech, Suzhou, Jiangsu, China) was added to each tube, and the mixture was vortexed for 2 ~ 3 seconds. The samples were then centrifuged at 400 × g for 5 minutes, and the supernatant was discarded. About 400 μL of PBS was added to each tube, followed by vortexing for 2 ~ 3 seconds to resuspend the cells. The samples were then analyzed on the flow cytometer, and the expression of immune cell markers was determined based on fluorescence intensity. All data were collected and analyzed using FlowJo software (FlowJo LLC, Ashland, Oregon, USA).

### Flow cytometric analysis of serum cytokine expression

A cytokine multiplex assay kit based on immunofluorescence (CellGene Biology, Nanchang, Jiangxi, China) was used to detect the levels of 12 cytokines in the serum of the MMT group and HC1, including IL-1β/2/4/5/6/8/10/12p70/17A (IL-1β/2/4/5/6/8/10/12p70/17A), tumor necrosis factor-α, and interferon-α/γ (IFN-α/γ). Quantitative detection was performed using a BD FACSCanto II flow cytometer (Becton, Dickinson & Company, Franklin Lakes, New Jersey, USA). Serum samples were mixed with fluorescent antibodies and capture microspheres, then incubated at room temperature and protected from light for 2.5 hours. Following incubation, 1 mL of PBS (NCM Biotech, Suzhou, Jiangsu, China) was added to each tube, vortexed for 2 ~ 3 seconds, and centrifuged at 400 × g for 5 minutes. The supernatant was discarded, and 200 μL of PBS was added to each tube, vortexed for 2 ~ 3 seconds, and analyzed on the flow cytometer. Cytokine expression levels were calculated based on the mean fluorescence intensity.

### Hematological routine testing of participants

Clinical blood samples from the MMT group and HC1 were subjected to routine blood tests. Blood routine analysis was performed using the Sysmex XN1500 automated hematology analyzer (Sysmex Corporation, Kobe, Japan). The instrument accurately measured multiple parameters, including red blood cell count, hemoglobin concentration, hematocrit, mean corpuscular volume, mean corpuscular hemoglobin (MCH), MCH concentration, white blood cell count (WBC), WBC differential count, platelet count, and mean platelet volume. All samples were processed according to standard operating procedures to ensure the accuracy and reliability of the test results.

### Genotyping

A total of 36 SNPs were selected, including cytochrome P450 family 3 subfamily A member 4 (rs2246709), cytochrome P450 family 2 subfamily B member 6 (rs707265, rs2279345, rs1038376, rs10403955, rs8100458, rs7250991, rs8192719, rs10853744, and rs16974799), ATP-binding cassette subfamily B member 1 (rs1045642 and rs1128503), opioid receptor mu 1 (OPRM1) (rs562859, rs1799971, rs2075572, rs495491, rs589046, rs10457090, rs3192723, rs6912029, and rs6902403), opioid receptor delta 1 (rs678849 and rs204076), catechol-O-methyltransferase (COMT) (rs4680, rs737866, and rs933271), brain-derived neurotrophic factor (rs6265), dopamine receptor D2 (rs1799978, rs1076560, and rs1799978), nerve growth factor beta (rs2239622), opioid receptor kappa 1 (rs997917, rs6985606, and rs6473797), cannabinoid receptor 1 (rs806368), and cholinergic receptor nicotinic alpha 2 subunit (rs2565055), in accordance with previous studies about MMT. About 5 mL of peripheral blood from the MMT group and HC1 were collected in tubes coated with ethylene diamine tetraacetic acid. The experimental process was divided into three steps: First, gDNA was extracted with MagBeads Blood DNA Extraction Kit; second, for the quality control of extracted gDNA, the integrity of genomic DNA was detected by agarose gel electrophoresis, and DNA concentration was measured by a microspectrophotometer. Finally, the genotypes of SNP were detected by the general fluorescent probe method using the Bio-Rad CFX96 fluorometer. All experimental procedures were strictly following the manufacturer’s standard protocol. To improve genotyping accuracy, a researcher who was blind to the addiction status of each participant genotyped each sample.

### Statistical analyses

The demographic, neuropsychological, hematological, and genotypic characteristics were analyzed using IBM SPSS Statistics v.26.0. Gender and matrimony were tested by R × C chi-square tests. As for continuous data, normally distributed data manifested by mean ± standard deviation were tested by two-sample *t*-tests; skewed data manifested by median (25% confidence interval ~ 75% confidence interval) were tested by Mann–Whitney tests.

### Neuroimaging data processing

All scans were processed using the PANDA software, which is a MATLAB toolbox that integrates MRIcron, FSL, and Diffusion Toolkit. PANDA employs an automated pipeline for processing cerebral diffusion images through the following steps: (1) Image format conversion: The DICOM files were transformed to Nifti format via the dcm2nii tool in MRIcron. (2) Brain extraction and mask estimation: Non-brain tissues were removed, and a brain mask was generated to isolate brain anatomy. (3) Redundant image regions were cropped to reduce memory usage, followed by correction of motion artifacts and eddy current-induced distortions. (4) DTI metric calculation: The calculation of diffusion indices was accomplished via diffusion tensor fitting implemented in FSL DTIFit, including FA, MD, AD, and RD. (5) Spatial normalization: The derived diffusion metrics were spatially normalized to the Montreal Neurological Institute (MNI) space through FSL FNIRT. A Tract-Based Spatial Statistics (TBSS) analysis was performed on PANDA to exclude voxels in the cerebrospinal fluid and gray matter, followed by thresholding of the mean FA skeleton using the typical 0.2 cutoff value. DTI metric images were generated in all subjects and subsequently computed within the WM skeleton as part of the statistical analysis.

### TBSS analysis

Voxel-wise statistical analyses between the MMT group and HC1, OUD1, and HC2, as well as OUD2 and HC2, were conducted by permutation-based two-sample *t*-statistics implemented through the FSL randomize command within the WM skeleton. A permutation-based paired *t*-statistic was used to conduct voxel-wise statistical analyses between OUD1 and OUD2. In total, 5,000 permutations and threshold-free cluster enhancement were applied to obtain a multiple-comparison corrected *p* value. Using a family-wise error rate correction, WM voxels reaching a significance threshold of *p* < 0.05 (corrected) were deemed statistically significant.

### Correlation and mediation analysis

Within the MMT group, Pearson (both variables are normally distributed) and Spearman (either variable is skewed) partial correlation analyses were used to explore relationships between significant DTI indexes and neuropsychological scales, MMT and heroin conditions, routine blood test indicators, T- or B-cell fine classification, regulatory T cells (Tregs), and inflammatory cytokines, by taking age, gender, BMI, education, FTND, AUDIT, matrimony, and differential genotypes (including rs6902403 and rs933271) as covariates. Then, heatmaps were created by calculating the correlation coefficient through ‘corrplot’ package in R 4.2.1. To investigate potential biological mechanisms underlying the relationship between significant DTI metrics and psychological symptoms, we conducted mediation analysis via the ‘mediation’ package in R 4.2.1 to explore whether there were mediating effects among hematologic immune cells or cytokines-DTI indexes-psychological scores, taking above 7 demographic characteristics and genotypic signatures as covariates. The variance inflation factor (VIF) was computed using multiple linear regression to discover potential multicollinearity among independent variables and covariates. Multiple comparisons were corrected by the Bonferroni method.

### Mendelian randomization

Recently, MR has emerged as a prevalent methodological tool in epidemiological analysis by employing genetic instrumental variables (IVs) to assess exposure-outcome causal relationships (Chen et al., 2024). To further exclude the influence of other factors (e.g., society, environment, and heredity) on opioid addiction and validate the results of TBSS, two-sample MR analyses were performed between OUD (exposure) and WM-related IDPs (outcome) (Rosoff et al., 2025). Genome-wide association study (GWAS) data of 384 IDPs (including FA, MD, L1, L2, L3, ICVF, ISOVF, and ODI; the number of each IDP category was 48) performed in a European-ancestry sample from the UK Biobank (Smith et al., 2021), as well as GWAS data of OUD from the FinnGen database (finngen_R12_F5_OPIOIDS.gz). We selected SNPs associated with OUD at a genome-wide significance threshold (*p* < 5 × 10^−6^) as candidate IVs. F-statistics were calculated to test the strength of these selected IVs (*F* > 10) (Mu, Dang, & Luo, 2024). Then, we used five MR techniques, including weighted model, inverse variance weighting, MR pleiotropy residual sum and outlier, weighted median, and MR-Egger regression to investigate their causal relationships. Finally, in sensitivity analyses (*p*-value set at 0.1), we carried out Cochran’s *Q* test to assess IVs’ heterogeneity of both MR-Egger and inverse-variance weighted (IVW) results and performed the MR Egger regression test to investigate horizontal pleiotropy. ‘Two-sample MR’ (version 0.5.6) package in R version 4.2.1. (R Foundation for Statistical Computing, Vienna, Austria) was used for all analyses to analyze causal relationships between OUDs and IDPs. Finally, to discover potential biological mechanisms underlying causal inference, IVs derived from MR analysis with significant results were used to conduct Gene Ontology enrichment analysis via WebGestalt 2024 (Elizarraras et al., 2024).

## Results

### Demographic, neuropsychological, and hematological characteristics

A comparison of clinical characteristics between the MMT group and HC1 was exhibited in Table 1 and Supplementary Tables S1–S4. As shown in Table 1, there was no statistically significant difference between the MMT group and HC1 in demographic characteristics except for matrimony (*p* < 0.001). OUD1 showed a lower education level and a higher FTND score than HC2. As for neuropsychological manifestations, the MMT group had significantly higher Barratt-total/no-planning/attention, HAMD, HAMA, PSQI, and ISI scores than HC1 (Supplementary Table S1). Despite being within normal levels, the MMT group had a lower level of platelet and platelet critical volume than that of the HC1 group (Supplementary Table S2). Supplementary Tables S3 and S4 indicated that MMT group had greater peripheral immunoinflammatory levels involving immune cells (including CD3 + CD4 + CD25hiCD127dim, CD3 + HLA-DR + CD38+, CD3 + CD4 + CD38 + HLA-DR+, CD3 + CD8 + CD38 + HLA-DR+, and CD3 + CD8 + CD38+), as well as inflammatory cytokines (including IL-1β/2/4/5/6/8/10/12p70 and IFN-α/γ); the proportion of CD19 + CD20 + CD27 + lgD+ and CD3 + HLA-DR+ of MMT group was significantly lower than HC1. The visualization of differences in immune cells was exhibited in Supplementary Figure S1.

**Table 1.** Demographic characteristics between the MMT group and HC1

Characteristics	MMT group (*n* = 53)	HC1 (*n* = 53)	*Z*/*χ* ^2^	*p*-value
Age (years)	51 (45, 56.5)	53 (46.5, 58.5)	*Z* = –0.066	0.947
Gender (male/female)	37/16	38/15	*χ* ^2^ = 0.046	0.831
BMI (kg/m^2^)	23.12 (19.71, 25.49)	23.88 (22.04, 26.08)	*Z* = –1.618	0.106
Education (years)	12 (9, 12)	9 (9, 12)	*Z* = 1.691	0.091
FTND (score)	4 (3, 6)	4 (0, 8)	*Z* = –0.229	0.819
AUDIT (score)	0 (0, 0)	0 (0, 0)	*Z* = –0.619	0.536
Matrimony (yes/no)	28/25	50/3	*χ* ^2^ = 23.491	0.000*
MMT condition				
Age of MMT initiation (years)	41.06 ± 1.31	/		
Duration of MMT (years)	10.05 ± 0.68	/		
Average MMT dosage (mg/day)	20 (10, 35)	/		
Heroin condition				
Age of first heroin use (years)	23 (20, 28)	/		
Duration of heroin use (years)	10 (5, 15)	/		
Age of regular heroin use (years)	25 (21, 30)	/		
Duration of regular heroin use (years)	8 (4, 14.5)	/		
Total dosage of regular heroin use (g)	521.43 (179.90, 2737.50)	/		
Times of heroin relapse	2 (1, 4)	/		
Duration of social reintegration (years)	9 (5, 14)	/		
/				

Characteristics	OUD1 group (*n* = 68)	HC2 (*n* = 56)	*Z*/*χ* ^2^	*p*-value
Age (years)	41.94 ± 7.18	41.88 ± 8.58	*t* = 0.046	0.963
Gender (male/female)	40/28	40/16	*χ* ^2^ = 2.131	0.144
Education (years)	8.33 ± 3.30	10.71 ± 2.71	*t* = −4.293	0.000*
FTND (score)	5 (5, 7)	0 (0, 2)	*Z* = 5.169	0.000*
AUDIT (score)	3 (0, 5)	0 (0, 5.75)	*Z* = –1.403	0.161
Heroin condition				
Age of first heroin use (years)	24 (21, 31.75)	/		
Duration of heroin use (years)	16.25 (9.88, 20.33)	/		
Total dosage of regular heroin use (g)	2907 (848, 4709)	/		
Abstinent duration at baseline (days)	30 (13.25, 77.5)	/		
OUD2: Abstinent duration at follow-up (days)	313.5 (227, 386.25)	/		

*Note:* *p < 0.05. Normal distributed data are in the form of mean ± SD (standard deviation). Skewed data are exhibited by median (25% confidence interval ~ 75% confidence interval). AUDIT, Alcohol Use Disorders Identification Test; BMI, body mass index; FTND, Fagerstrom Test for Nicotine Dependence; HC1, healthy controls – cohort 1; HC2, healthy controls – cohort 2; OUD1, abstinent heroin users at baseline; OUD2, abstinent heroin users at around 10-month follow-up; MMT, methadone maintenance treatment.

### Diffusion metrics

**Figure 2.**
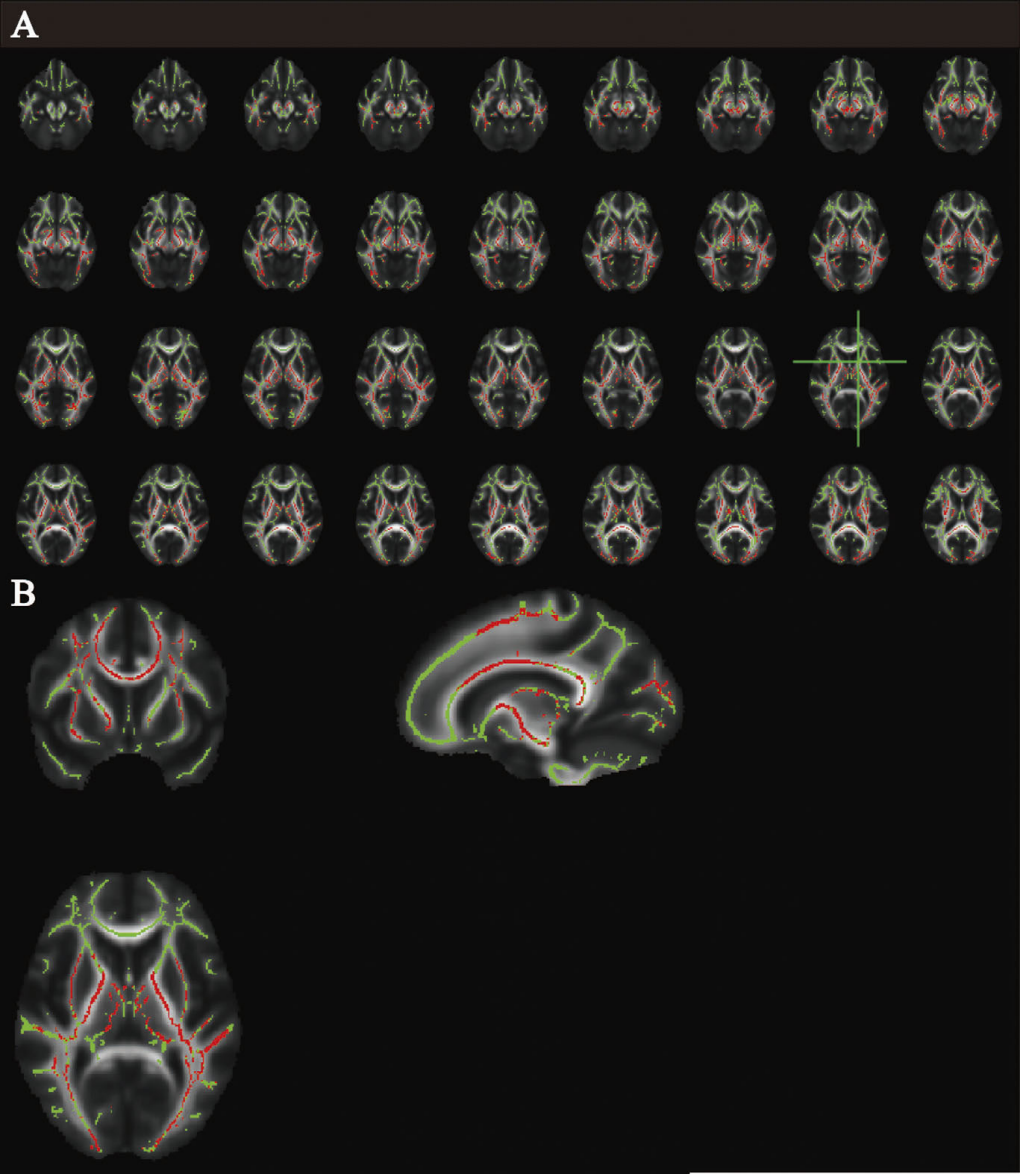
Significant results of tract-based spatial statistics between the methadone maintenance treatment (MMT) group and healthy controls-cohort 1 (HC1). (A) Higher mean diffusivity and radial diffusivity occurred in the same white matter (WM) regions of the MMT group; (B) the most aberrant WM was visualized at the green axis level.

**Figure 3.**
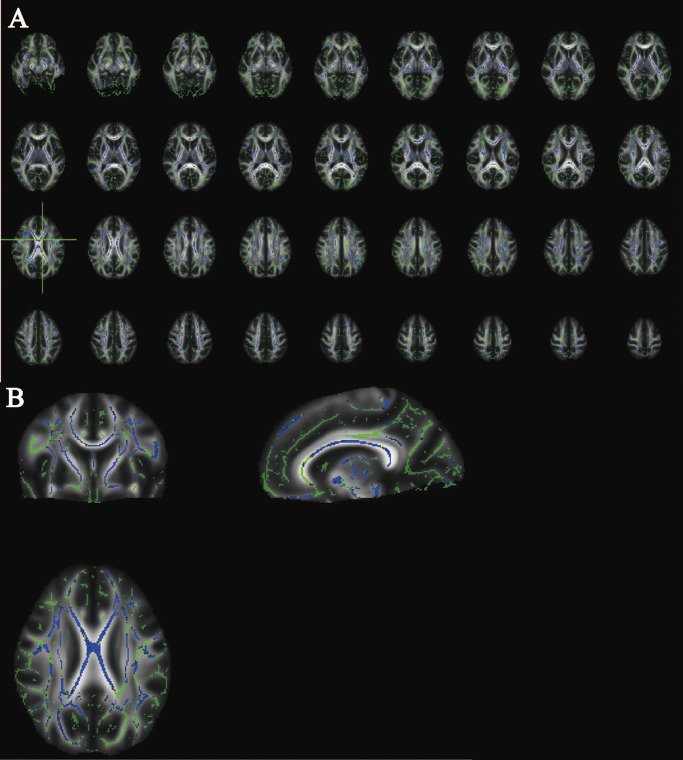
Significant results of tract-based spatial statistics between abstinent heroin users at baseline (OUD1) and healthy controls-cohort 2 (HC2)/abstinent heroin users at around 10-month follow-up (OUD2). (A) Lower mean diffusivity and axial diffusivity were displayed in the same white matter (WM) regions of OUD1; (B) the most aberrant WM was visualized at the green axis level.

Compared to HC1, TBSS analyses revealed higher MD and RD measurements in the MMT group in a wide range of cerebral regions (Figure 2). Compared to OUD2 and HC2, OUD1 showed lower MD and AD measurements (Figure 3). There was no significant difference in DTI metrics between OUD2 and HC2. Details of the significant results were exhibited in Supplementary Table S5. A total of six clusters with mostly overlapping brain regions were found to be abnormal. The bilateral uncinate fasciculus was a specific abnormal brain area for the MMT group. Bilateral fornix (cres)/Stria terminalis and right superior fronto-occipital fasciculus were specific abnormal brain areas for the OUD1 group. The peak MNI coordinate represents the specific cerebral locations where regions exhibited the highest level of MD or RD. Peak *T*-value is a statistic calculated by voxel-wise statistical analysis. According to genotypic stratification within the MMT group, OPRM1 rs6902403 TT/CT and COMT rs933271 CT/CC genotypes, respectively, displayed higher RD_Cluster2_ and MD_(Cluster1)_ than corresponding CC and TT genotypes (Supplementary Table S6).

### Correlation and mediation outcomes

This study focused on the relationships of diffusion metrics with other clinical characteristics. As shown in Supplementary Figures S2 and S3, within the MMT group, MD_(Cluster1)_ and RD_(Cluster2)_ were positively correlated with PSQI scores (both *p* < 0.05/30, Bonferroni corrected; *r* = 0.52, 0.59, respectively) and IFN-γ (both *p* < 0.05/24, Bonferroni corrected; *r* = 0.63, 0.55, respectively). There were positive correlations between MD_(Cluster1)_ and RD_Cluster2_ and ISI (both *p* < 0.01, uncorrected; *r* = 0.43, 0.49, respectively), as well as CD19+ (both *p* < 0.05, uncorrected; *r* = 0.50, 0.45, respectively). As exhibited in Table 2, mediation analyses demonstrated that there were significant forward mediating effects among IFN-γ – MD_(Cluster1)_ or RD_Cluster2_ – PSQI (both total effect *p*-values < 0.05/8, Bonferroni corrected). Average causal mediation effects and average direct effects were also significant (all *p* < 0.05). All VIFs were close to 1, demonstrating that mediation results were not impacted by multicollinearity. In addition, there is moderate-to-intensive significance in positive associations among heroin craving, PSQI, ISI, HAMD, and HAMA, as well as among times of heroin relapse, total dosage of regular heroin use, duration of heroin use, and duration of regular heroin use. The correlations between blood routine examination measurements and neuroimaging features are shown in Supplementary Figure S4.

**Table 2.** Significant results of mediation analysis among serum immunity-related genotypes-MD/RD-PSQI/ISI within the MMT group

Mediation pathways	ACME		ADE		Total effect	
	Estimate (95% CI)	*p*-value	Estimate (95% CI)	*p*-value	Estimate (95% CI)	*p*-value
CD19+ − MD_(Cluster 1)_ - PSQI	0.25 (0.001,0.78)	0.046*	−0.24 (−0.82, 0.34)	0.39	0.01 (−0.56, 0.58)	0.97
CD19+ − RD_(Cluster 2)_ - PSQI	0.31 (0.001, 0.80)	0.008*	−0.30 (−0.83, 0.23)	0.25	0.01 (−0.56, 0.58)	0.97
CD19+ − MD_(Cluster 1)_ - ISI	0.28 (−0.10, 0.80)	0.06	−0.33 (−1.03, 0.37)	0.33	−0.05 (−0.72, 0.63)	0.88
CD19+ − RD_(Cluster 2)_ - ISI	0.31 (−0.095, 0.78)	0.05	−0.35 (−1.03, 0.32)	0.29	−0.05 (−0.72, 0.63)	0.88
IFN-γ − MD_(Cluster 1)_ - PSQI	3.36 (0.43, 6.24)	0.01*	4.30 (0.65, 7.95)	0.02*	7.66 (4.77, 10.55)	0.000^#^
IFN-γ − RD_(Cluster 2)_ - PSQI	3.44 (0.81, 6.10)	0.004*	4.22 (0.74, 7.70)	0.02*	7.66 (4.77, 10.55)	0.000^#^
IFN-γ − MD_(Cluster 1)_ - ISI	0.15 (−1.34, 1.47)	0.68	−1.56 (−5.72, 2.59)	0.44	−1.41 (−5.39, 2.57)	0.47
IFN-γ − RD_(Cluster 2)_ - ISI	0.15 (−1.39, 1.23)	0.65	−1.56 (−5.70, 2.58)	0.44	−1.41 (−5.39, 2.57)	0.47

*Note:* ^#^*p* < (0.05/8), Bonferroni corrected; *p < 0.05. ACME, Average causal mediation effect; ADE, average direct effect; CI, confidence interval; ISI, Insomnia Severity Index; IFN-γ, interferon-γ; MD, mean diffusivity; MMT, methadone maintenance treatment; PSQI, Pittsburgh Sleep Quality Index; RD, radial diffusivity.

### Causal relationships

**Figure 4.**
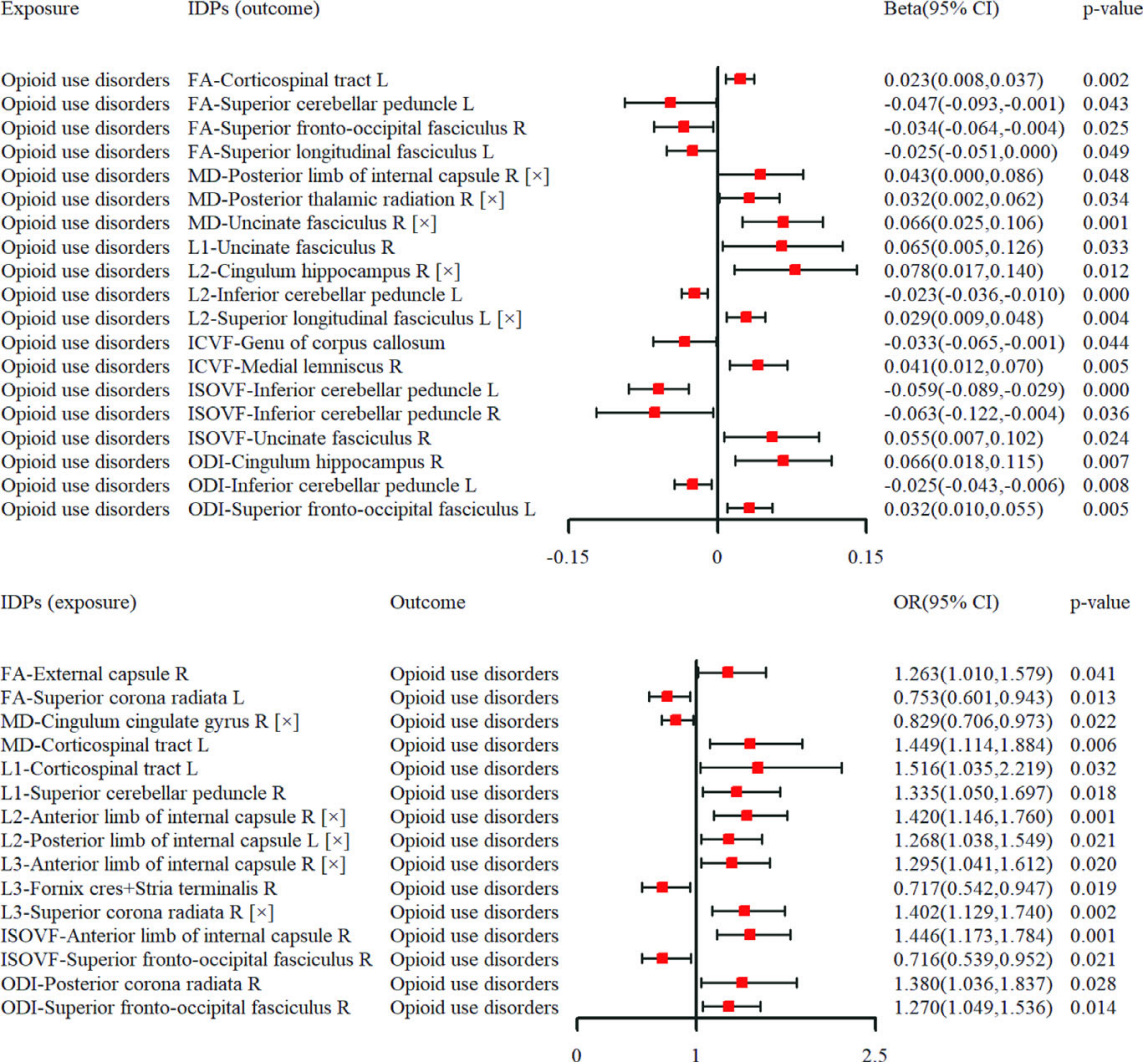
Causal relationships between opioid use disorders (exposure) and white matter-related imaging-derived phenotypes (IDPs) in forward (A) and inverse (B) Mendelian randomization analysis. ‘[×]’ indicated intersected results between MR and tract-based spatial statistics analysis. CI, confidence interval; FA, fractional anisotropy; ICVF, intracellular volume fraction; ISOVF, isotropic volume fraction; MD, mean diffusivity; OR, odds ratio; ODI, Orientation Dispersion Index.

We further intersected the results of MR and TBSS analyses (MMT group vs. HC1). As displayed in Supplementary Table S7 and Figure 4, forward MR analyses found positive relationships between OUD and MD (in right posterior thalamic radiation [PTR], right posterior limb of internal capsule [IC], and right uncinate fasciculus [UF]), as well as L2 (in left superior longitudinal fasciculus [SLF]). Inverse MR analyses indicated positive associations between L2 (in right anterior limb of IC and left posterior limb of IC), as well as L3 (in right anterior limb of IC and right superior corona radiata [SCR]) and OUD. These brain regions also occurred in the TBSS results. As shown in Supplementary Table S8, all significant causal results had no existing heterogeneity and horizontal pleiotropy (all *p* > 0.1). In supplementary files, Supplementary Table S9 showed utilized IVs following clumping and harmonization; Supplementary Table S10 showed full results of MR analyses via five approaches; and Supplementary Table S11 showed the results of heterogeneity and horizontal pleiotropy in MR analysis. Genetic enrichment analysis indicates a significant genetic enrichment in ‘response to axon injury’ and ‘regulation of tube size’, implying that they mainly underlie hereditary mechanisms of causal inference (Supplementary Figure S5).

## Discussion

One of the primary challenges in treating opioid addiction with methadone is the incomplete framework of the neural signature of neuropsychological symptoms that were intimately correlated with heroin craving. In the current study, WM alterations of different withdrawal statuses were mapped. We quantified the WM damage correlated with insomnia and uncovered its underlying biological foundation through multimodal datasets. For the first time, we reported that IFN-γ might act as a critical initiator in insomnia-related WM alterations during MMT. These findings bridge cytokine disturbance to macroscale cerebral structure, shedding light on potential therapeutic targets for optimizing MMT strategy.

Initially, an extensively aberrant WM integrity represented by higher MD and RD corresponding to insomnia symptoms was unraveled in OUDs with MMT. However, OUD1 exhibited lower MD and AD than both HC2 and OUD2. To sum up, our DTI study depicted different fate trajectories of WM microstructure after heroin dependence: short-term withdrawal (OUD1, median: 30 days) might present acute neurotoxicity/inflammation-related axonal or dendritic damages reflected by decreased MD and AD; long-term withdrawal (OUD2, median: 313.5 days) underscored significant plasticity recovery of neural microstructure manifested by MD and AD normalization; although long-term MMT can effectively help to maintain condition stability and inhibit opioid craving, it is accompanied by extensive signs of density decline of neuron components (e.g. axons and dendrites) exhibited by higher MD and RD, suggesting that long-term μ-opioid receptor excitation stimulated by methadone may pose a unique challenge to WM health. Intersected results between MR and TBSS (MMT group vs HC1) indicated specific neuroimaging markers for opioid-related brain injury, particularly in IC, right PTR, right UF, left SLF, and right SCR, whose disrupted fiber membrane integrity predicted a higher risk of sleep disorders, especially in insomnia (Bresser et al., 2020, 2023; Lima Santos, Soehner, Ladouceur, & Versace, 2025; Uy et al., 2023). Interestingly, we further detected moderate-to-intensive associations between PSQI or ISI and heroin craving, HAMD, as well as HAMA, indicating that there might be insomnia-craving/depression/anxiety interactions in the MMT group. Given previous studies elucidating the influence of insomnia on opioid relapse (Bolling, Cardoni, & Arnedt, 2025; Hochheimer et al., 2025), we also proposed that improvement of sleep quality would be a vital step to suppress the recurrence of opioid usage. The neuroimaging features of WM disorganization provided subjective diagnostic indicators for MMT-related insomnia. Besides, OPRM1 rs6902403 and COMT rs933271 genotypes might mediate the susceptibility of WM injury.

Then, this study revealed significant dysregulation of the peripheral immune system in MMT patients manifested by increased levels of a wide range of pro/anti-inflammatory cytokines and altered proportions of T/B-cell subsets, in accordance with previous studies (Butelman et al., 2023; Chan et al., 2015; Zhu et al., 2023). The MMT group exhibited significantly elevated levels of multiple peripheral immunoinflammatory cell subsets, including highly activated total T cells (CD3^+^HLA-DR^+^CD38^+^) and activated CD4^+^/CD8^+^ T-cell subsets (CD3^+^CD4^+^CD38^+^HLA-DR^+^, CD3^+^CD8^+^CD38^+^HLA-DR^+^, and CD3^+^CD8^+^CD38^+^), which indicated chronic immune/inflammatory activation. Concomitantly, the elevated frequency of CD3^+^CD4^+^CD25ʰⁱCD127ᵈⁱᵐ Tregs likely represents a compensatory immunomodulatory response to the persistent inflammation induced by methadone treatment (Longhi, Mieli-Vergani, & Vergani, 2021). Collectively, these observations point to a state of systemic T-cell hyperactivation and immune homeostasis imbalance in MMT patients, characterized by compensatory Treg elevation alongside the activation of pro-inflammatory T-cell subsets. This immunoinflammatory profile may underlie the neuropsychological comorbidities (e.g., insomnia and depression) and immune dysfunction associated with long-term MMT, highlighting these flow cytometric markers as potential biomarkers for evaluating immune status in OUD patients undergoing MMT. In contrast to the increase in proinflammatory T-cell subpopulation, there was a reduction in the proportion of CD19 + CD20 + CD27 + IgD+ B cells in the MMT group, which were unswitched memory B cells. This phenomenon might portend B-cell exhaustion or enhanced differentiation into plasma cells in a chronic inflammatory state. Due to B cells universally suppressing excessive immune responses by secreting anti-inflammatory factors, B-/T-cell imbalance would exacerbate the uncontrollability of neuroinflammation (Lee et al., 2024). Besides, IL-4 is a well-known driver of microglia or macrophage to an anti-inflammatory phenotype (M2) (Liu et al., 2024). The IL-10 and IL-4 expansion in this study might be a compensatory regulation, while their levels are inadequate to offset the overall pro-inflammatory state.

Subsequently, ‘IFN-γ-WM damage-insomnia’ axis could be a potential targeted pathway in sustaining MMT-related neuropsychological homeostasis. IFN-γ, secreted by activated T cells (mainly CD4+ Th1 cells and CD8+ cytotoxic T lymphocytes) and natural killer cells, is a multifunctional cytokine belonging to the type II IFN family and plays an important role in psychiatric disorders like OUDs, Alzheimer’s disease, and schizophrenia (Halstead et al., 2023; Li et al., 2025; Z. Yin et al., 2023). In 2023, a seminal study published in *Cell* by Zhu et al. revealed opioid-activated systemic hypoxia in peripheral circulation, which triggered the vulnerability of Tregs (Zhu et al., 2023). This pathophysiological cascade stimulated IFN-γ release that mediated opioid-induced synaptic remodeling in the nucleus accumbens via blood–brain barrier and subsequent withdrawal symptoms (Zhu et al., 2023). Impaired immune homeostasis (e.g., dysregulated pro-inflammatory cytokine signaling) plays a critical role in the pathogenesis of chronic insomnia. Zahra Aghelan et al. reported elevated serum IFN-γ levels in patients with chronic insomnia (Aghelan et al., 2023). Elevated IFN-γ modulates the relationship between sleep disturbance and depressive mood: Specifically, the correlation between sleep disturbance and depressive mood is stronger in individuals with higher IFN-γ levels than in those with lower IFN-γ levels (Piber et al., 2023). Consistent with findings by Jiahui Yin et al., these results collectively suggest that dysregulated IFN-γ signaling acts as a key mediating mechanism in insomnia comorbid with psychiatric disorders (Yin et al., 2023). Notably, pharmacological blockade of IFN-γ signaling attenuated withdrawal symptoms in morphine-dependent mouse models through modulating neuroimmune crosstalk (Zhu et al., 2023). It further elucidated that IFN-γ coordinates this process via downstream immunomodulatory pathways linking peripheral hypoxia-stimulated immune response to central neural adaptation. Beyond synaptic remodeling of neurons, our study showed that the microstructure of other neural elements, encompassing axons and dendrites, can be affected by IFN-γ, which can exacerbate central nervous system damage by provoking a toxic environment and suppressing axonal growth (Garcia et al., 2023). Hence, our work, based on multidisciplinary human information, first elucidates the associations among peripheral IFN-γ, WM imaging signature, and insomnia in long-term OUDs with MMT, which offers novel insights into therapeutic targets.

Currently, it is the first study harnessing the largest sample sizes and multiple cohorts to investigate the impacts of MMT on WM organization and incorporate multimodal datasets to explore biological mechanisms of MMT-related insomnia. However, several limitations should be addressed. First, although it can acknowledge reversible WM recovery to normality following 10-month non-MMT abstinence and then demonstrate that the increase in MD and RD might be caused by MMT instead of heroin use history, disconnection of OUD1 and MMT attenuated the robustness of the TBSS result. Second, plasma neuron-specific enolase and S100 calcium-binding protein B are commonly peripheral brain damage markers and deserve to be considered in future studies to further prove cerebral WM disruption (Demirci et al., 2023). Third, due to the influence of inadequate statistical power, threshold effects, and unmeasured confounding factors (e.g. environmental pressure), greater sample size and more qualitative or quantitative measurements of other factors were required to be included to investigate the relationships between MD_(Cluster1)_ or RD_(Cluster2)_ and craving, depression, and anxiety, which would provide more clinical significance for WM lesions within MMT patients. Ultimately, beyond FA, MD, AD, and RD, more diffusion indexes like ODI need to be obtained to uncover delicate microstructural alterations of WM.

In conclusion, our results recognize aberrant WM organization in OUDs with MMT, which is related to peripheral IFN-γ elevation and insomnia. In view of positive associations between insomnia and heroin craving/depression/anxiety manifestations (Liu et al., 2025; Zhang et al., 2024), it might be endowed with vital meaning to target INF-γ therapy for MMT patients with insomnia. Excitingly, from a neuroimaging perspective, this study further confirms the importance of IFN-γ in the research field of OUD withdrawal treatment, following the article published by Zhu et al.

## Supporting information

10.1017/S0033291726103614.sm001 Yang et al. supplementary material Yang et al. supplementary material

## Data Availability

The datasets used and/or analyzed during the current study are available from the corresponding author upon reasonable request.

## Supplementary material

The supplementary material for this article can be found at http://doi.org/10.1017/S0033291726103614.
