# Supplementary material for: Crosstalk of peripheral cytokine-white matter alteration-insomnia during methadone maintenance treatment
Source: Psychol Med. 2026 Apr 27;56:e113. doi: 10.1017/S0033291726103614 (PMC13125936; doi:10.1017/S0033291726103614)
Supplement: Yang et al. supplementary material [file S0033291726103614sup001.zip › Supplementary Materials_monochrome.docx]

Supplementary Materials for

Crosstalk of peripheral immunity-white matter alteration-insomnia during methadone maintenance treatment

**This PDF file includes:**

Materials and Methods

Figures S1 to S5

Tables S1 to S6

Materials and Methods

Methods

**Detailed Software Versions and Parameters.**

| **Section** | **Tool/Software** | **Version** | **Description** |
| --- | --- | --- | --- |
| Immune cell percentage | BD FACSCanto II flow cytometer | - | Immune cell quantitative detection |
|  | FlowJo | 10.8.1 | Immune cell population analyzing |
| Serum cytokine expression | BD FACSCanto II flow cytometer | - | Serum cytokine quantitative detection |
|  | FlowJo | 10.8.1 | Serum cytokine population analyzing |
| Routine blood tests | Sysmex Corporation, Kobe, Japan | - | Blood routine analysis |
| SNP genotypes | Bio-Rad CFX96 fluorometer | - | Genotyping |
| White matter integrity analyses | MAGNETOM Skyra, Siemens Healthcare, Erlangen, Germany | - | DTI scanning |
|  | MRIcron | v1.0.20190902 | DICOM to Nifti format |
|  | FSL | 6.0.7.7 | Tract-based spatial statistics |
|  | FSLeyes | 1.15.0 | Result visualization of tract-based spatial statistics |
| Data analyses | SPSS | 26.0 | Two-sample t tests; paired t tests; chi-square tests; Mann-Whitney tests |
| Correlation analyses | R("corrplot" package) | 4.2.1 | Pearson or Spearman partial correlation analyses |
| Causal relationship analyses | R("Two sample MR" package) | 4.2.1 | Mendelian randomization |
| Biological mechanisms | WebGestalt 2024 | - | Gene Ontology (GO) enrichment analysis |

Materials

**Antibodies and reagents for cyTOF**

| **Reagent or Resource** | **Source** | **Identifier** | **Clone** | **Label** |
| --- | --- | --- | --- | --- |
| **Antibodies** | | |  |  |
| Hu HLA-DR | BD Biosciences | 555811 | L243 | FITC |
| Hu CD28 | BD Biosciences | 555729 | CD28.2 | PE |
| Hu CD4 | BD Biosciences | 566924 | SK3 | PerCP |
| Hu CD45RA | BD Biosciences | 568557 | HI100 | PE-CY7 |
| Hu CD8 | BD Biosciences | 555369 | RPA-T8 | APC |
| Hu CD3 | BD Biosciences | 557832 | SK7 | APC-Cy7 |
| Hu CD38 | BD Biosciences | 562444 | HIT2 | BV421 |
| Hu CD45 | BD Biosciences | 563204 | HI30 | BV510 |
| Hu CD45RA | BD Biosciences | 555488 | HI100 | FITC |
| Hu CD127 | BD Biosciences | 557938 | HIL-7R-M21 | PE |
| Hu CD25 | BD Biosciences | 555434 | M-A251 | APC |
| Hu IgD | BD Biosciences | 555778 | IA6-2 | FITC |
| Hu CD27 | BD Biosciences | 560612 | M-T271 | PerCP-Cy5.5 |
| Hu CD19 | BD Biosciences | 557835 | SJ25C1 | PE-CY7 |
| Hu CD20 | BD Biosciences | 559776 | 2H7 | APC |


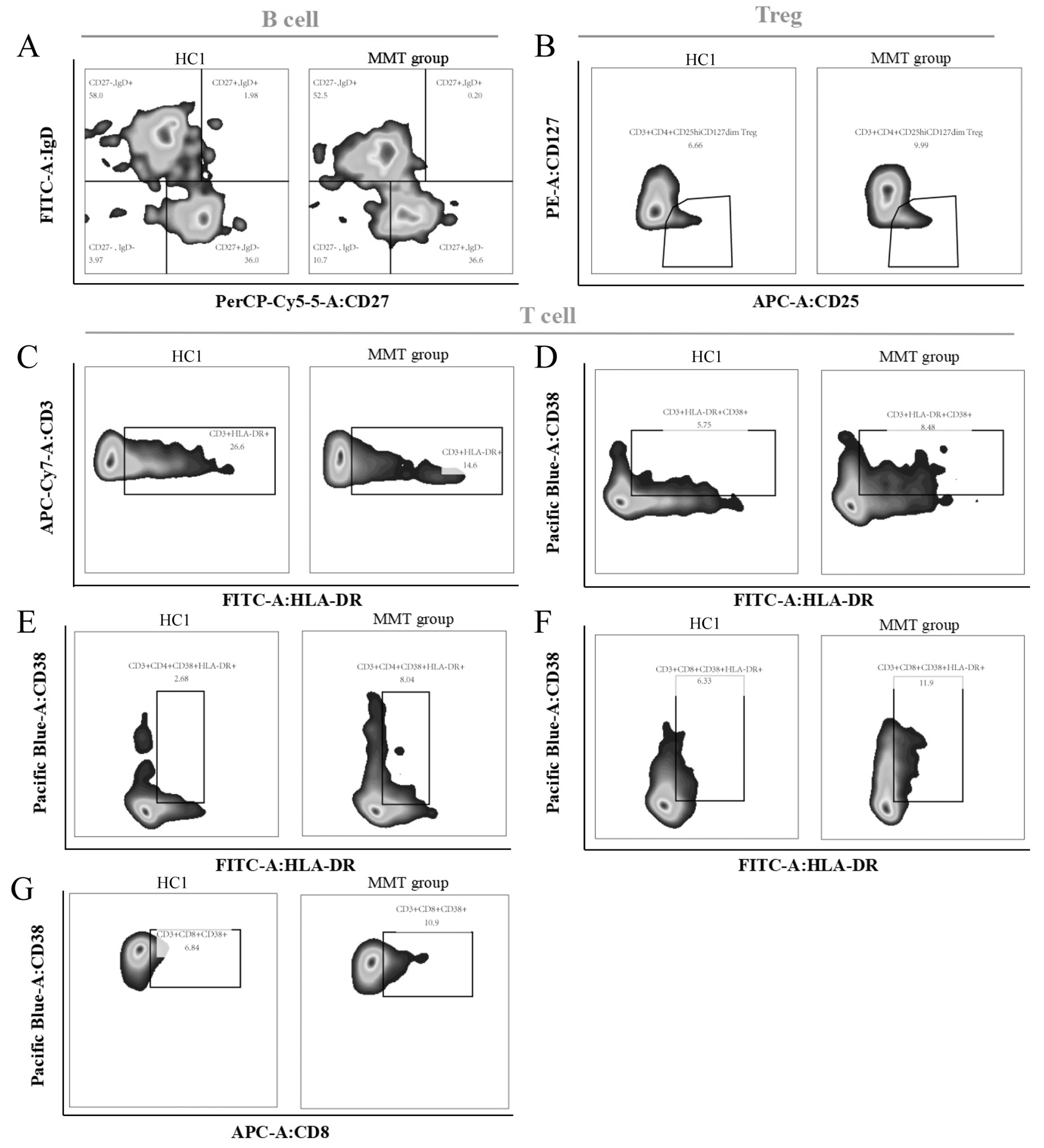


Figure S1. Comparison of different immune cell subsets between healthy controls-cohort1 (HC1) and methadone maintenance treatment (MMT) group. (A) Compared to HC1, subpopulation analysis of B cells showed that CD19+CD20+CD27+lgD+ cell population was significantly reduced in MMT group; (B) The CD3+CD4+CD25hiCD127dim cell population was significantly elevated in MMT group; (C) CD3+HLA-DR+ cell population was significantly decreased in MMT group; (D) CD3+ HLA-DR+CD38+ cell population was increased in MMT group; (E-G) Further subdivision of T cells showed that CD3+CD4+CD38+HLA-DR+ in CD4 T cells, CD3+CD8+CD38+HLA-DR+ and CD3+CD8+CD38+ subsets in CD8T cells were significantly increased in MMT group.


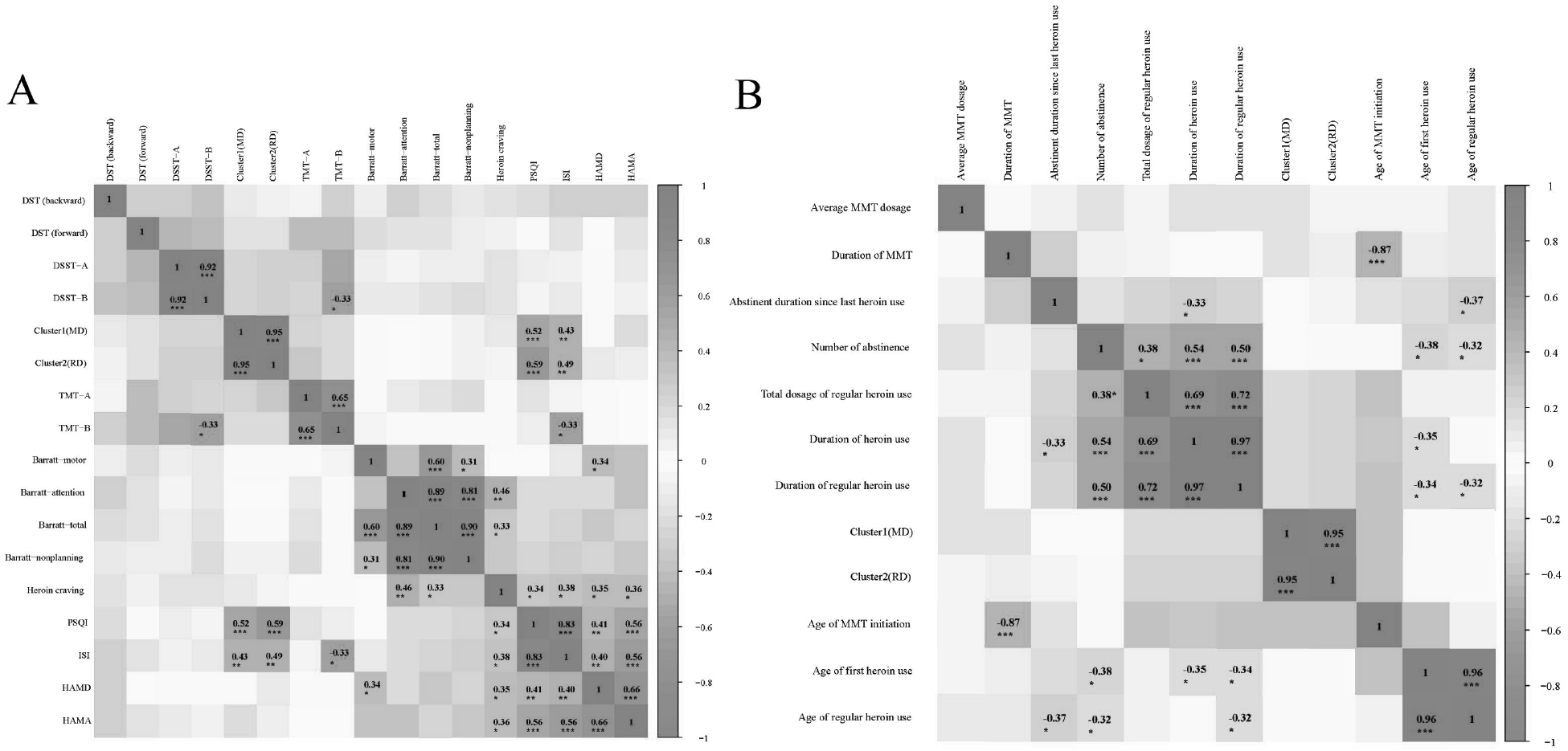


Figure S2. Heatmaps of correlations between neuroimaging features and neuropsychological scores (A) as well as clinical measurements (B) within methadone maintenance treatment (MMT) group. DST, digit span test; HAMA, Hamilton anxiety scale; HAMD, Hamilton depression scale; HC1, healthy controls-cohort1; ISI, insomnia severity index; MD, mean diffusivity; PSQI, Pittsburgh sleep quality index; RD, radial diffusivity; DSST-A/B, symbol substitution test-A/B; TMT-A/B, trail-making test A/B. *p<0.05, **p<0.01, ***p<0.001.


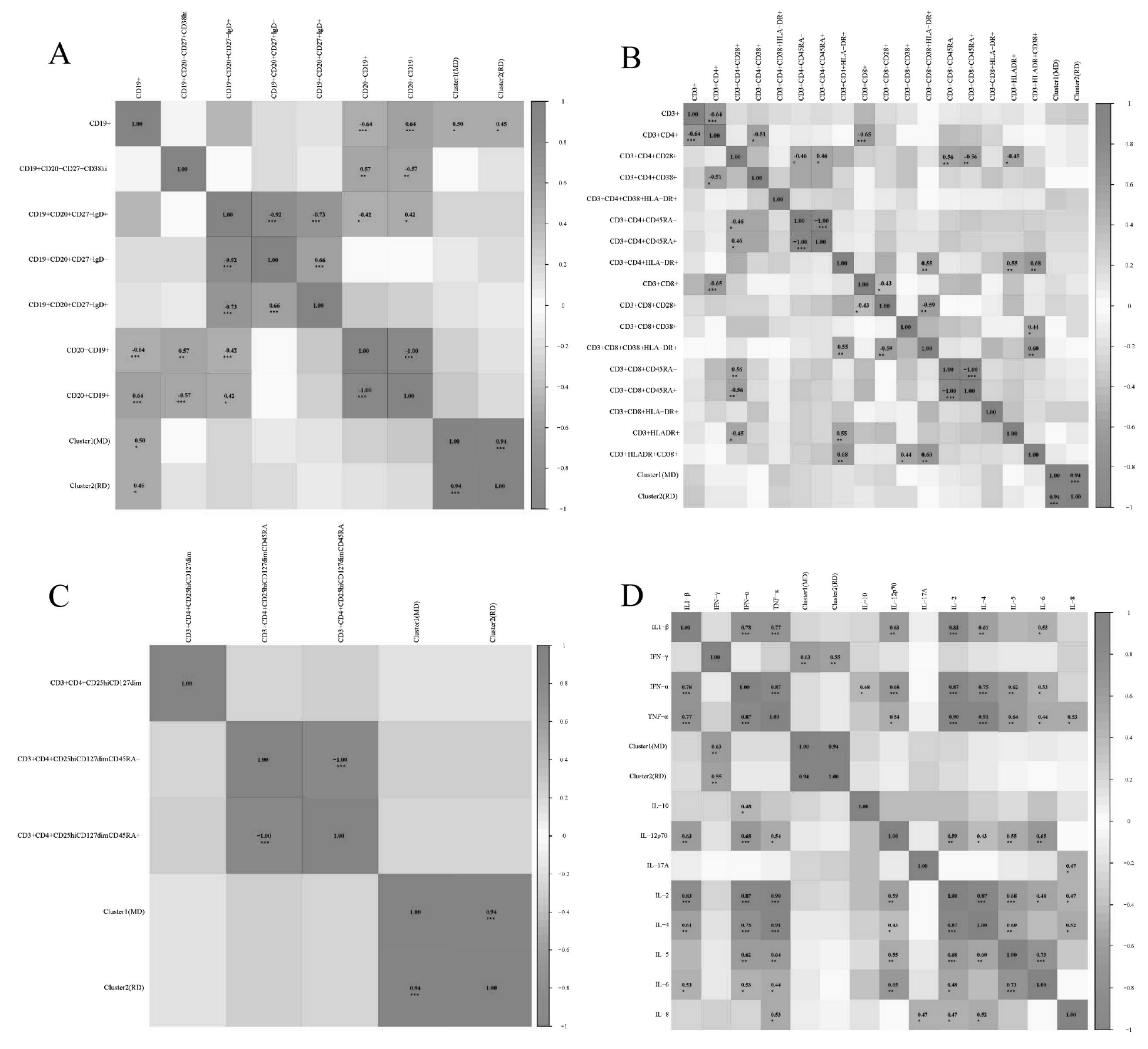


Figure S3. Heatmaps of correlations between neuroimaging features and B cells (A), T cells (B), regulatory T cells (C), as well as inflammatory factors (D) within methadone maintenance treatment (MMT) group. IL-1β/2/4/5/6/8/10/12p70/17A, interleukin-1β/2/4/5/6/8/10/12p70/17A; IFN-α, interferon-α; IFN-γ, interferon-γ; MMT, methadone maintenance treatment; TNF-α, tumor necrosis factor-α. *p<0.05, **p<0.01, ***p<0.001.


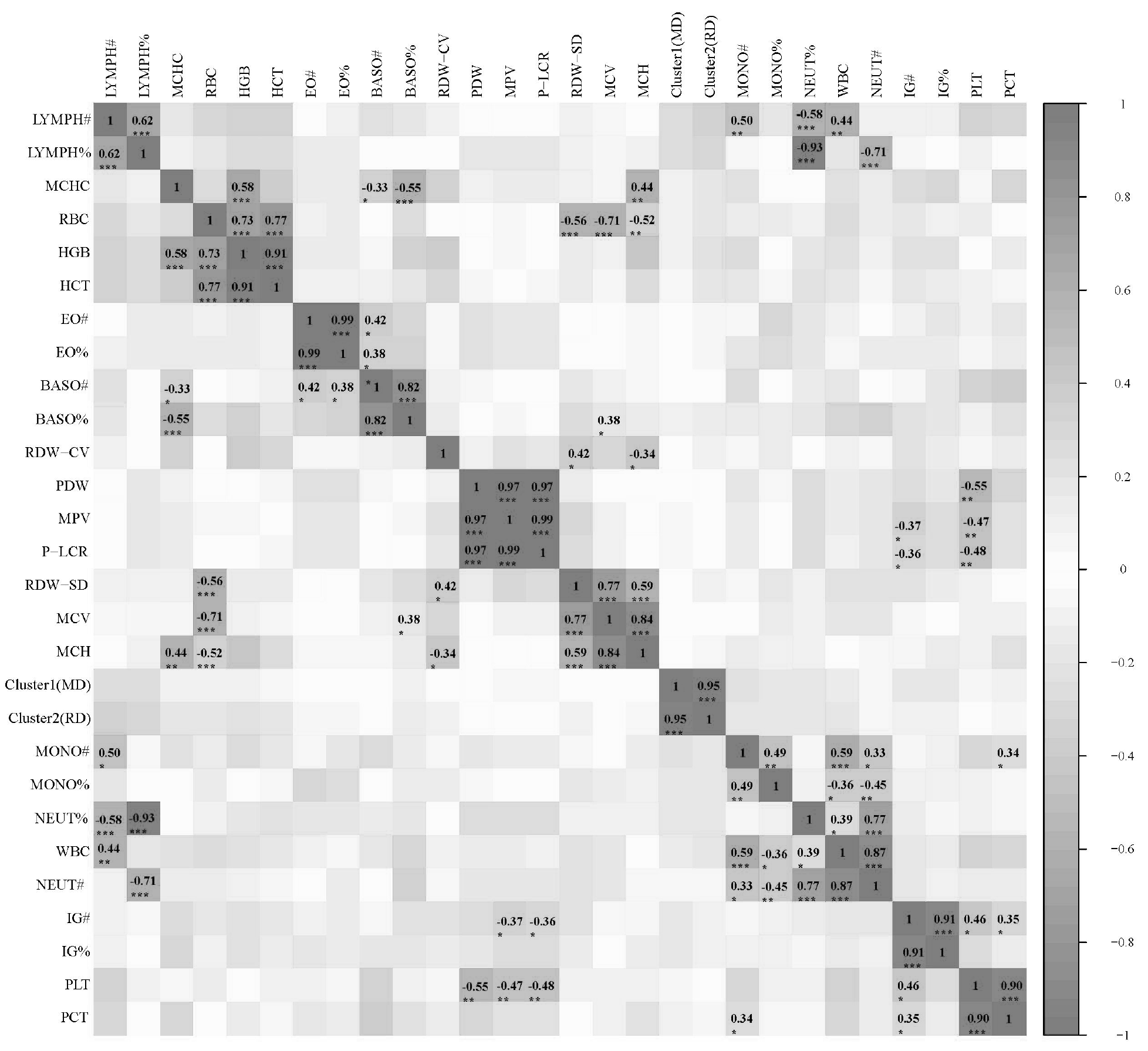


Figure S4. Heatmaps of correlations between neuroimaging features and blood routine examination measurements within methadone maintenance treatment group. BASO#, basophil absolute count; BASO%, basophil percentage; EO#, eosinophil absolute count; EO%, eosinophil percentage; HC1, healthy controls-cohort1; HCT, hematocrit; HGB, hemoglobin; IG#, immature granulocyte count; IG%, immature granulocyte percentage; LYMPH#, lymphocyte absolute count; LYMPH%, lymphocyte percentage; MCH, mean corpuscular hemoglobin; MCHC, mean corpuscular hemoglobin concentration; MCV, mean corpuscular volume; MD, mean diffusivity; MPV, mean platelet volume; MONO#, monocyte absolute count; MONO%, monocyte percentage; NEUT#, neutrophil absolute count; NEUT%, neutrophil percentage; PLT, platelet; PCT, platelet critical volume; PDW, platelet distribution width; P-LCR, platelet large cell ratio; RBC, red blood cell; RD, radial diffusivity; RDW-CV, red cell distribution width-coefficient of variation; RDW-SD, red cell distribution width-standard deviation; WBC, white blood cell. *p<0.05, **p<0.01, ***p<0.001.


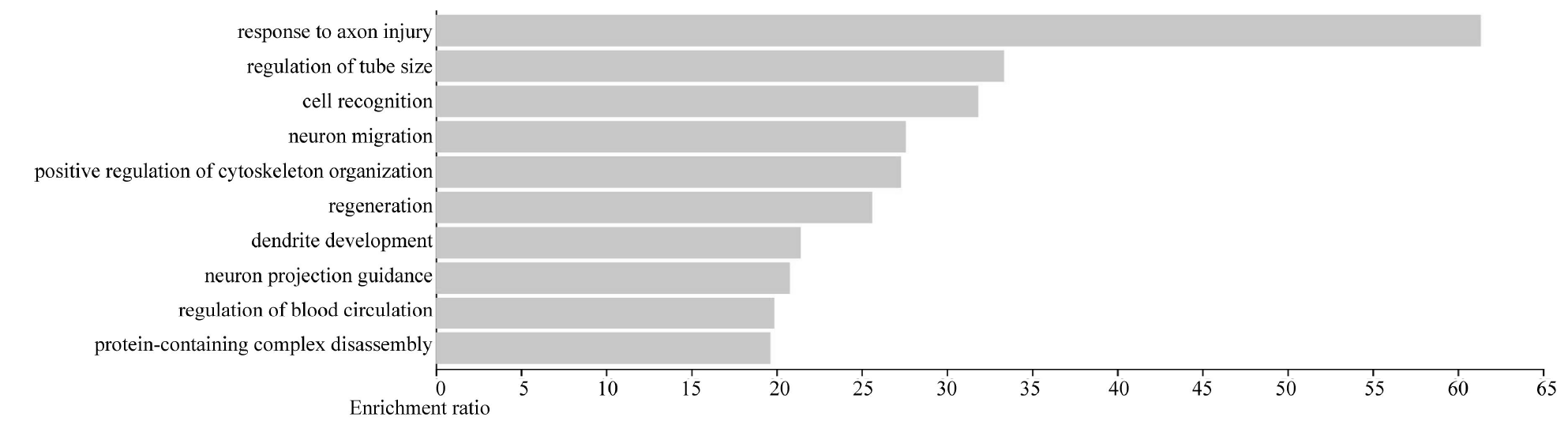


Figure S5. Gene Ontology enrichment analysis of instrumental variables used in Mendelian randomization analysis with significant results.

**Table S1.** Summary of neuropsychological characteristics in MMT group and HC1.

| Neuropsychological scales | MMT group (n=53) | HC1 (n=53) | t/Z | p-value |
| --- | --- | --- | --- | --- |
| Barratt-total | 76.15±21.08 | 64.17±15.87 | t=3.306 | 0.001^#^ |
| Barratt-no-planning | 28(20.5, 33.5) | 23(19, 26) | Z=4.355 | 0.000^#^ |
| Barratt-motor | 22(16, 29.5) | 18(13, 24) | Z=0.579 | 0.563 |
| Barratt-attention | 26.51±21.72 | 8.90±6.56 | t=3.155 | 0.002^#^ |
| TMT-A (s) | 37.17(29.29, 48.99) | 40.94(32.76, 52.75) | Z=-1.507 | 0.132 |
| TMT-B (s) | 95.35(69.69, 113.82) | 94.56(74.24, 111.91) | Z=-0.186 | 0.852 |
| DSST-A | 27(20, 33) | 22(19, 26.5) | Z=1.942 | 0.052 |
| DSST-B | 54(38.5, 65.5) | 46(35.5, 54) | Z=1.956 | 0.051 |
| DST (forward) | 13(12, 14) | 13(12, 14) | Z=-0.364 | 0.716 |
| DST (backward) | 6(4, 7.5) | 6(4, 8) | Z=-0.131 | 0.869 |
| HAMD | 9(6.5, 16.5) | 2(0, 4) | Z=6.936 | 0.000^#^ |
| HAMA | 7(3, 12) | 2(0, 3) | Z=5.652 | 0.000^#^ |
| PSQI | 11(6, 14) | 4(2, 7) | Z=5.439 | 0.000^#^ |
| ISI | 8(3, 15) | 2(1, 5) | Z=5.055 | 0.000^#^ |
| Heroin craving | 32(25, 40) | / | | |

^#^p<(0.05/15), Bonferroni corrected. Normal distributed data are in the form of mean ± SD (standard deviation). Skewed data are exhibited by median (25% confidence interval~75% confidence interval). DST, digit span test; DSST-A/B, symbol substitution test-A/B; HAMA, Hamilton anxiety scale; HAMD, Hamilton depression scale; HC1, healthy controls-cohort1; ISI, insomnia severity index; PSQI, Pittsburgh sleep quality index; TMT-A/B, trail-making test A/B.

**Table S2.** Summary of blood routine examination in MMT group and HC1.

| Routine blood test indicators | MMT group (n=44) | HC1 (n=44) | t/Z | p-value |
| --- | --- | --- | --- | --- |
| WBC (×10^9^/L) | 6.31±1.65 | 6.18±1.78 | t=0.352 | 0.726 |
| HGB (g/L) | 140.05±13.13 | 142.09±18.80 | t=-0.592 | 0.556 |
| RBC (×10^12^/L) | 4.59±0.41 | 4.84±0.72 | t=-2.062 | 0.042 |
| PLT (×10^9^/L) | 176.45±58.65 | 227.20±47.89 | t=-4.446 | 0.000^#^ |
| NEUT(×10^9^/L) | 3.42±1.31 | 3.36±1.14 | t=0.221 | 0.825 |
| NEUT (%) | 53.56±10.64 | 53.97±7.77 | t=-0.207 | 0.836 |
| LYMPH (×10^9^/L) | 2.10(1.75,2.59) | 2.04(1.63, 2.61) | Z=-0.204 | 0.839 |
| LYMPH (%) | 34.81±10.23 | 35.05±7.97 | t=-0.120 | 0.905 |
| EO (×10^9^/L) | 0.11(0.05, 0.20) | 0.16(0.10, 0.22) | Z=-1.701 | 0.089 |
| EO (%) | 1.90(1.10, 2.90) | 2.45(1.63, 4.13) | Z=-1.996 | 0.046 |
| BASO (×10^9^/L) | 0.035±0.015 | 0.039±0.017 | t=-1.214 | 0.228 |
| BASO (%) | 0.57±0.27 | 0.65±0.27 | t=-1.312 | 0.193 |
| MONO (×10^9^/L) | 0.52(0.40, 0.61) | 0.43(0.33, 0.60) | Z=-2.001 | 0.045 |
| MONO (%) | 8.30(7.50, 9.90) | 7.40(6.13, 7.98) | Z=-3.138 | 0.002 |
| IG (×10^9^/L) | 0.01(0.01, 0.02) | 0.01(0.01, 0.02) | Z=-0.937 | 0.349 |
| IG (%) | 0.20(0.10, 0.30) | 0.20(0.10, 0.30) | Z=-0.805 | 0.421 |
| HCT (%) | 42.75±3.25 | 43.71±5.04 | t=-1.059 | 0.293 |
| MCV (fL) | 93.55(90.40, 95.35) | 92.85(89.50, 95.20) | Z=-1.110 | 0.267 |
| MCH (pg) | 30.85(29.70, 31.80) | 30.10(29.40, 30.88) | Z=-1.816 | 0.069 |
| MCHC (g/L) | 327.25±11.93 | 324.34±10.87 | t=1.195 | 0.235 |
| RDW-CV (%) | 12.90(12.40, 13.20) | 12.75(12.33, 13.45) | Z=-0.085 | 0.932 |
| RDW-SD | 44.40±3.07 | 44.49±4.08 | t=-0.114 | 0.909 |
| MPV (fL) | 10.39±0.95 | 10.62±0.89 | t=-1.116 | 0.268 |
| PCT (%) | 0.19(0.14, 0.22) | 0.24(0.22, 0.28) | Z=-4.877 | 0.000^#^ |
| P-LCR (%) | 28.16±7.97 | 29.42±7.13 | t=-0.762 | 0.448 |
| PDW (fL) | 12.27±2.25 | 12.43±1.94 | t=-0.358 | 0.721 |

^#^p<(0.05/26), Bonferroni corrected. Normal distributed data are in the form of mean ± SD (standard deviation). Skewed data are exhibited by median (25% confidence interval~75% confidence interval). BASO#, basophil absolute count; BASO%, basophil percentage; EO#, eosinophil absolute count; EO%, eosinophil percentage; HC1, healthy controls-cohort1; HCT, hematocrit; HGB, hemoglobin; IG#, immature granulocyte count; IG%, immature granulocyte percentage; LYMPH#, lymphocyte absolute count; LYMPH%, lymphocyte percentage; MCH, mean corpuscular hemoglobin; MCHC, mean corpuscular hemoglobin concentration; MCV, mean corpuscular volume; MPV, mean platelet volume; MONO#, monocyte absolute count; MONO%, monocyte percentage; NEUT#, neutrophil absolute count; NEUT%, neutrophil percentage; PLT, platelet; PCT, platelet critical volume; PDW, platelet distribution width; P-LCR, platelet large cell ratio; RBC, red blood cell; RDW-CV, red cell distribution width-coefficient of variation; RDW-SD, red cell distribution width-standard deviation; WBC, white blood cell.

**Table S3.** Summary of B cell, T cell, and T regulatory cell (Treg) in MMT group and HC1.

| Immune cell (%) | MMT group (n=37) | HC1 (n=40) | t/Z | p-value |
| --- | --- | --- | --- | --- |
| B cell | | | | |
| CD19+ | 9.00(7.20, 11.75) | 12.50(10.55, 14.38) | Z=-3.120 | 0.002 |
| CD19+CD20+ | 97.50(94.80, 98.40) | 97.70(95.15, 98.80) | Z=-0.709 | 0.478 |
| CD19+CD20- | 2.45(1.62, 5.21) | 2.275(1.19, 4.84) | Z=0.704 | 0.482 |
| CD19+CD20+CD27-lgD+ | 71.27±12.43 | 66.72±11.93 | t=1.639 | 0.105 |
| CD19+CD20+CD27+lgD+ | 0.80(0.40, 1.50) | 1.72(1.15, 3.76) | Z=-3.452 | 0.001^#^ |
| CD19+CD20+CD27+lgD- | 18.20(12.55, 25.70) | 25.00(19.73, 30.18) | Z=-2.488 | 0.013 |
| CD19+CD20-CD27+CD38hi | 0.16(0.11, 0.29) | 0.15(0.11, 0.28) | Z=0.582 | 0.561 |
| T regulatory cell | | | | |
| CD3+CD4+CD25hiCD127dim | 8.91(6.84, 10.35) | 6.56(5.46, 7.19) | Z=4.44 | 0.000^#^ |
| CD3+CD4+CD25hiCD127dimCD45RA- | 84.44±7.19 | 88.77±5.51 | t=-2.975 | 0.004 |
| CD3+CD4+CD25hiCD127dimCD45RA+ | 15.56±7.19 | 11.23±5.51 | t=2.975 | 0.004 |
| T cell | | | | |
| CD3+ | 65.55±11.57 | 62.99±9.98 | t=1.04 | 0.302 |
| CD3+CD4+ | 49.17±12.71 | 50.12±12.58 | t=-0.33 | 0.741 |
| CD3+CD8+ | 35.75±9.73 | 38.76±12.22 | t=-1.188 | 0.238 |
| CD3+HLA-DR+ | 15.20(13.15, 17.40) | 21.25(15.90, 29.65) | Z=-3.661 | 0.000^#^ |
| CD3+HLA-DR+CD38+ | 7.03(6.16, 8.09) | 5.20(3.49, 6.40) | Z=3.931 | 0.000^#^ |
| CD3+CD4+CD28+ | 95.70(93.25, 98.35) | 89.85(84.73, 95.13) | Z=3.039 | 0.002 |
| CD3+CD4+CD38+ | 10.80(9.09, 13.85) | 8.41(6.13, 12.48) | Z=2.519 | 0.012 |
| CD3+CD4+CD38+HLA-DR+ | 6.19(4.43, 7.39) | 2.67(1.92, 3.44) | Z=5.149 | 0.000^#^ |
| CD3+CD4+CD45RA- | 66.80±11.54 | 66.38±14.36 | t=0.147 | 0.883 |
| CD3+CD4+CD45RA+ | 33.15±11.53 | 33.61±14.34 | t=-0.155 | 0.877 |
| CD3+CD8+HLA-DR+ | 19.17±4.78 | 21.88±9.14 | t=-1.648 | 0.105 |
| CD3+CD8+CD45RA- | 63.44±12.37 | 65.28±15.01 | t=-0.582 | 0.563 |
| CD3+CD8+CD45RA+ | 36.26±12.33 | 34.39±14.91 | t=0.597 | 0.552 |
| CD3+CD8+CD38+HLA-DR+ | 9.05(6.82, 12.10) | 5.49(3.35, 8.43) | Z=4.165 | 0.000^#^ |
| CD3+CD8+CD38+ | 11.30(9.11, 14.45) | 6.73(5.72, 9.32) | Z=4.930 | 0.000^#^ |
| CD3+CD8+CD28+ | 56.22±16.64 | 50.53±19.45 | t=1.374 | 0.174 |
| CD3+CD4+HLA-DR+ | 12.30±3.59 | 13.25±5.73 | t=-0.874 | 0.385 |

^#^p<(0.05/27), Bonferroni corrected. Normal distributed data are in the form of mean ± SD (standard deviation). Skewed data are exhibited by median (25% confidence interval~75% confidence interval). MMT, methadone maintenance treatment; HC1, healthy controls-cohort1.

**Table S4.** Summary of inflammatory factors in MMT group and HC1.

| Inflammatory factors level(%) | MMT group (n=37) | HC1 (n=40) | t/Z | p-value |
| --- | --- | --- | --- | --- |
| IL-1β | 4.82 (3.07, 6.63) | 1.54(1.14, 1.93) | Z=5.915 | 0.000^#^ |
| IL-2 | 2.81(2.66, 3.19) | 1.97(1.68, 2.55) | Z=4.566 | 0.000^#^ |
| IL-4 | 2.46(2.13, 3.12) | 1.93(1.62, 2.30) | Z=4.282 | 0.000^#^ |
| IL-5 | 0.94(0.87, 1.08) | 0.65(0.57, 0.78) | Z=4.882 | 0.000^#^ |
| IL-6 | 5.66(3.96, 7.02) | 3.66(3.16, 5.46) | Z=2.953 | 0.003^#^ |
| IL-8 | 27.18(22.43, 37.13) | 7.26(6.10, 8.11) | Z=7.153 | 0.000^#^ |
| IL-10 | 6.03(4.96, 8.55) | 3.58(2.71, 5.59) | Z=4.340 | 0.000^#^ |
| IL-12p70 | 2.65(2.08, 3.43) | 1.98(1.64, 2.44) | Z=3.472 | 0.001^#^ |
| IL-17A | 7.12(4.90, 9.14) | 4.67(3.14, 8.35) | Z=2.052 | 0.040 |
| TNF-α | 1.76(1.20, 2.78) | 1.20(0.88, 2.30) | Z=1.822 | 0.069 |
| IFN-α | 2.29(1.87, 2.64) | 1.52(1.30, 1.84) | Z=5.177 | 0.000^#^ |
| IFN-γ | 1.10(0.85, 1.47) | 0.79(0.46, 1.10) | Z=3.216 | 0.001^#^ |

^#^p<(0.05/12), Bonferroni corrected. Skewed data are exhibited by median (25% confidence interval~75% confidence interval). HC1, healthy controls-cohort1; IL-1β/2/4/5/6/8/10/12p70/17A, interleukin-1β/2/4/5/6/8/10/12p70/17A; IFN-α, interferon-α; IFN-γ, interferon-γ; MMT, methadone maintenance treatment; TNF-α, tumor necrosis factor-α.

**Table S5.** Tract‐based spatial statistics analysis of FA, MD, and RD between MMT group and HC1 as well as among OUD1, OUD2, and HC2.

| Modality Contrast | Cluster number (number of voxels) | Peak MNI coordinates | | | Peak T‐value | Common anatomical region | Specific anatomical region | p-value |
| --- | --- | --- | --- | --- | --- | --- | --- | --- |
|  |  | X | Y | Z |  |  |  |  |
| *MD [MMT (n=49) > HC1 (n=51)]* | Cluster 1 (42569) | 62 | 106 | 90 | 6.011 | Genu/body/splenium of corpus callosum, fornix (column and body of fornix), bilateral cerebral peduncle, bilateral anterior/posterior limb of internal capsule, retrolenticular part of internal capsule, bilateral superior/anterior/posterior corona radiata, bilateral posterior thalamic radiation (include optic radiation), bilateral external capsule, bilateral superior longitudinal fasciculus, bilateral sagittal stratum (include inferior longitudinal fasciculus and inferior fronto-occipital fasciculus), bilateral cingulum (cingulate gyrus) | Bilateral uncinate fasciculus | 0.000^#^ |
| *RD [MMT (n=49) > HC1 (n=51)]* | Cluster 2 (41153) | 56 | 109 | 97 | 5.683 |  |  | 0.000^#^ |
| *MD [OUD1 (n=68) < HC2 (n=56)]* | Cluster 3 (25026) | 115 | 107 | 81 | 4.590 |  | Bilateral fornix(cres)/Stria terminalis; right superior fronto-occipital fasciculus (could be a part of anterior internal capsule) | 0.000^#^ |
| *AD [MMT (n=68) < HC2 (n=56)]* | Cluster 4 (13884) | 115 | 107 | 83 | 3.692 |  |  | 0.000^#^ |
| *MD [OUD1 (n=61) < OUD2 (n=61)]* | Cluster 5 (19168) | 77 | 109 | 57 | 6.865 |  |  | 0.000^#^ |
| *AD [OUD1 (n=61) < OUD2 (n=61)]* | Cluster 6 (17074) | 113 | 105 | 71 | 8.559 |  |  | 0.000^#^ |

^#^p<0.05, Bonferroni corrected. FA, fractional anisotropy; HC1, healthy controls-cohort 1; HC2, healthy controls-cohort 2; OUD1, abstinent heroin users at baseline; OUD2, abstinent heroin users at around 10-month follow-up; MD, mean diffusivity; MMT, methadone maintenance treatment; MNI, Montreal Neurological Institute; RD, radial diffusivity.

**Table S6****.** Comparisons of DTI indexes stratified by SNP genotypes within MMT group.

| SNP sites | DTI indexes | Genotypes | N | Mean | SD | t | p value |
| --- | --- | --- | --- | --- | --- | --- | --- |
| rs6902403 | MD_(Cluster 1)_ | TT/CT | 32 | 7.60×10^-4^ | 2.04×10^-5^ | 1.667 | 0.102 |
|  |  | CC | 15 | 7.49×10^-4^ | 1.82×10^-5^ |  |  |
|  | RD_(Cluster 2)_ | TT/CT | 32 | 5.39×10^-4^ | 2.28×10^-5^ | 2.133 | 0.038^*^ |
|  |  | CC | 15 | 5.23×10^-4^ | 2.46×10^-5^ |  |  |
| rs933271 | MD_(Cluster 1)_ | TT | 18 | 7.49×10^-4^ | 1.91×10^-5^ | -2.133 | 0.038^*^ |
|  |  | CT/CC | 29 | 7.61×10^-4^ | 1.96×10^-5^ |  |  |
|  | RD_(Cluster 2)_ | TT | 18 | 5.28×10^-4^ | 2.37×10^-5^ | -1.251 | 0.218 |
|  |  | CT/CC | 29 | 5.37×10^-4^ | 2.44×10^-5^ |  |  |
| rs1038376 | MD_(Cluster 1)_ | AA | 31 | 7.53×10^-4^ | 2.06×10^-5^ | -1.320 | 0.194 |
|  |  | AT/TT | 16 | 7.61×10^-4^ | 1.87×10^-5^ |  |  |
|  | RD_(Cluster 2)_ | AA | 31 | 5.31×10^-4^ | 2.42×10^-5^ | -0.972 | 0.336 |
|  |  | AT/TT | 16 | 5.39×10^-4^ | 2.46×10^-5^ |  |  |
| rs10403955 | MD_(Cluster 1)_ | TT | 25 | 7.56×10^-4^ | 2.26×10^-5^ | -0.019 | 0.985 |
|  |  | GT/GG | 22 | 7.56×10^-4^ | 1.75×10^-5^ |  |  |
|  | RD_(Cluster 2)_ | TT | 25 | 5.35×10^-4^ | 2.62×10^-5^ | 0.255 | 0.800 |
|  |  | GT/GG | 22 | 5.33×10^-4^ | 2.25×10^-5^ |  |  |
| rs10853744 | MD_(Cluster 1)_ | TT/GT | 22 | 7.56×10^-4^ | 1.75×10^-5^ | 0.019 | 0.985 |
|  |  | GG | 25 | 7.56×10^-4^ | 2.26×10^-5^ |  |  |
|  | RD_(Cluster 2)_ | TT/GT | 22 | 5.33×10^-4^ | 2.25×10^-5^ | -0.255 | 0.800 |
|  |  | GG | 25 | 5.35×10^-4^ | 2.62×10^-5^ |  |  |
| rs16974799 | MD_(Cluster 1)_ | TT/CT | 21 | 7.57×10^-4^ | 1.73×10^-5^ | 0.311 | 0.757 |
|  |  | CC | 26 | 7.55×10^-4^ | 2.25×10^-5^ |  |  |
|  | RD_(Cluster 2)_ | TT/CT | 21 | 5.35×10^-4^ | 2.11×10^-5^ | 0.230 | 0.819 |
|  |  | CC | 26 | 5.33×10^-4^ | 2.70×10^-5^ |  |  |
| rs2279345 | MD_(Cluster 1)_ | TT/CT | 21 | 7.53×10^-4^ | 2.12×10^-5^ | -1.088 | 0.282 |
|  |  | CC | 26 | 7.59×10^-4^ | 1.92×10^-5^ |  |  |
|  | RD_(Cluster 2)_ | TT/CT | 21 | 5.29×10^-4^ | 2.66×10^-5^ | -1.223 | 0.228 |
|  |  | CC | 26 | 5.38×10^-4^ | 2.20×10^-5^ |  |  |
| rs707265 | MD_(Cluster 1)_ | AA/AG | 25 | 7.54×10^-4^ | 2.00×10^-5^ | -0.771 | 0.445 |
|  |  | GG | 22 | 7.59×10^-4^ | 2.04×10^-5^ |  |  |
|  | RD_(Cluster 2)_ | AA/AG | 25 | 5.31×10^-4^ | 2.54×10^-5^ | -0.798 | 0.429 |
|  |  | GG | 22 | 5.37×10^-4^ | 2.32×10^-5^ |  |  |
| rs7250991 | MD_(Cluster 1)_ | AA | 26 | 7.55×10^-4^ | 2.25×10^-5^ | -0.311 | 0.757 |
|  |  | CA/CC | 21 | 7.57×10^-4^ | 1.73×10^-5^ |  |  |
|  | RD_(Cluster 2)_ | AA | 26 | 5.33×10^-4^ | 2.70×10^-5^ | -0.230 | 0.819 |
|  |  | CA/CC | 21 | 5.35×10^-4^ | 2.11×10^-5^ |  |  |
| rs8100458 | MD_(Cluster 1)_ | TT | 15 | 7.56×10^-4^ | 1.50×10^-5^ | -0.157 | 0.876 |
|  |  | CT/CC | 32 | 7.57×10^-4^ | 2.24×10^-5^ |  |  |
|  | RD_(Cluster 2)_ | TT | 15 | 5.29×10^-4^ | 1.88×10^-5^ | -0.946 | 0.349 |
|  |  | CT/CC | 32 | 5.36×10^-4^ | 2.64×10^-5^ |  |  |
| rs8192719 | MD_(Cluster 1)_ | TT/CT | 22 | 7.56×10^-4^ | 1.75×10^-5^ | 0.019 | 0.985 |
|  |  | CC | 25 | 7.56×10^-4^ | 2.26×10^-5^ |  |  |
|  | RD_(Cluster 2)_ | TT/CT | 22 | 5.33×10^-4^ | 2.25×10^-5^ | -0.255 | 0.800 |
|  |  | CC | 25 | 5.35×10^-4^ | 2.62×10^-5^ |  |  |
| rs10457090 | MD_(Cluster 1)_ | AA | 41 | 7.54×10^-4^ | 1.93×10^-5^ | -2.025 | 0.086 |
|  |  | GA | 6 | 7.72×10^-4^ | 2.01×10^-5^ |  |  |
|  | RD_(Cluster 2)_ | AA | 41 | 5.31×10^-4^ | 2.26×10^-5^ | -1.763 | 0.085 |
|  |  | GA | 6 | 5.50×10^-4^ | 3.16×10^-5^ |  |  |
| rs2075572 | MD_(Cluster 1)_ | CC | 33 | 7.53×10^-4^ | 1.87×10^-5^ | -1.528 | 0.134 |
|  |  | GC/GG | 14 | 7.63×10^-4^ | 2.24×10^-5^ |  |  |
|  | RD_(Cluster 2)_ | CC | 33 | 5.31×10^-4^ | 2.23×10^-5^ | -1.271 | 0.210 |
|  |  | GC/GG | 14 | 5.41×10^-4^ | 2.82×10^-5^ |  |  |
| rs562859 | MD_(Cluster 1)_ | TT | 36 | 7.56×10^-4^ | 2.01×10^-5^ | -0.079 | 0.937 |
|  |  | CC/CT | 11 | 7.57×10^-4^ | 2.11×10^-5^ |  |  |
|  | RD_(Cluster 2)_ | TT | 36 | 5.34×10^-4^ | 2.43×10^-5^ | 0.249 | 0.805 |
|  |  | CC/CT | 11 | 5.32×10^-4^ | 2.555×10^-5^ |  |  |
| rs589046 | MD_(Cluster 1)_ | TT/CT | 8 | 7.65×10^-4^ | 2.26×10^-5^ | 1.328 | 0.191 |
|  |  | CC | 39 | 7.54×10^-4^ | 1.94×10^-5^ |  |  |
|  | RD_(Cluster 2)_ | TT/CT | 8 | 5.40×10^-4^ | 3.34×10^-5^ | 0.850 | 0.400 |
|  |  | CC | 39 | 5.32×10^-4^ | 2.23×10^-5^ |  |  |
| rs1799971 | MD_(Cluster 1)_ | AA | 18 | 7.54×10^-4^ | 2.11×10^-5^ | -0.515 | 0.609 |
|  |  | GA/GG | 29 | 7.57×10^-4^ | 1.98×10^-5^ |  |  |
|  | RD_(Cluster 2)_ | AA | 18 | 5.32×10^-4^ | 2.68×10^-5^ | -0.473 | 0.638 |
|  |  | GA/GG | 29 | 5.35×10^-4^ | 2.30×10^-5^ |  |  |
| rs6912029 | MD_(Cluster 1)_ | GT | 6 | 7.72×10^-4^ | 2.01×10^-5^ | 2.025 | 0.086 |
|  |  | GG | 41 | 7.54×10^-4^ | 1.93×10^-5^ |  |  |
|  | RD_(Cluster 2)_ | GT | 6 | 5.50×10^-4^ | 3.16×10^-5^ | 1.763 | 0.085 |
|  |  | GG | 41 | 5.31×10^-4^ | 2.26×10^-5^ |  |  |
| rs495491 | MD_(Cluster 1)_ | AA | 39 | 7.54×10^-4^ | 1.94×10^-5^ | -1.328 | 0.191 |
|  |  | GA/GG | 8 | 7.65×10^-4^ | 2.26×10^-5^ |  |  |
|  | RD_(Cluster 2)_ | AA | 39 | 5.32×10^-4^ | 2.23×10^-5^ | -0.850 | 0.400 |
|  |  | GA/GG | 8 | 5.40×10^-4^ | 3.34×10^-5^ |  |  |
| rs204076 | MD_(Cluster 1)_ | AA | 36 | 7.55×10^-4^ | 1.93×10^-5^ | -0.658 | 0.514 |
|  |  | AT/TT | 11 | 7.60×10^-4^ | 2.33×10^-5^ |  |  |
|  | RD_(Cluster 2)_ | AA | 36 | 5.33×10^-4^ | 2.46×10^-5^ | -0.482 | 0.632 |
|  |  | AT/TT | 11 | 5.37×10^-4^ | 2.43×10^-5^ |  |  |
| rs678849 | MD_(Cluster 1)_ | TT | 18 | 7.50×10^-4^ | 2.00×10^-5^ | -1.701 | 0.096 |
|  |  | CT/CC | 29 | 7.60×10^-4^ | 1.96×10^-5^ |  |  |
|  | RD_(Cluster 2)_ | TT | 18 | 5.27×10^-4^ | 2.25×10^-5^ | -1.617 | 0.113 |
|  |  | CT/CC | 29 | 5.38×10^-4^ | 2.47×10^-5^ |  |  |
| rs1076560 | MD_(Cluster 1)_ | AA/AC | 33 | 7.57×10^-4^ | 2.10×10^-5^ | 0.550 | 0.585 |
|  |  | CC | 14 | 7.54×10^-4^ | 1.84×10^-5^ |  |  |
|  | RD_(Cluster 2)_ | AA/AC | 33 | 5.35×10^-4^ | 2.43×10^-5^ | 0.362 | 0.719 |
|  |  | CC | 14 | 5.32×10^-4^ | 2.51×10^-5^ |  |  |
| rs1800497 | MD_(Cluster 1)_ | AA/GA | 32 | 7.57×10^-4^ | 2.13×10^-5^ | 0.373 | 0.711 |
|  |  | GG | 15 | 7.55×10^-4^ | 1.80×10^-5^ |  |  |
|  | RD_(Cluster 2)_ | AA/GA | 32 | 5.34×10^-4^ | 2.46×10^-5^ | 0.198 | 0.844 |
|  |  | GG | 15 | 5.33×10^-4^ | 2.44×10^-5^ |  |  |
| rs1799978 | MD_(Cluster 1)_ | TT | 28 | 7.54×10^-4^ | 1.82×10^-5^ | -0.928 | 0.358 |
|  |  | CT/CC | 19 | 7.60×10^-4^ | 2.27×10^-5^ |  |  |
|  | RD_(Cluster 2)_ | TT | 28 | 5.30×10^-4^ | 2.11×10^-5^ | -1.277 | 0.208 |
|  |  | CT/CC | 19 | 5.39×10^-4^ | 2.80×10^-5^ |  |  |
| rs6473797 | MD_(Cluster 1)_ | TT | 16 | 7.53×10^-4^ | 2.09×10^-5^ | -0.860 | 0.394 |
|  |  | CT/CC | 31 | 7.58×10^-4^ | 1.98×10^-5^ |  |  |
|  | RD_(Cluster 2)_ | TT | 16 | 5.30×10^-4^ | 2.27×10^-5^ | -0.837 | 0.407 |
|  |  | CT/CC | 31 | 5.36×10^-4^ | 2.52×10^-5^ |  |  |
| rs997917 | MD_(Cluster 1)_ | TT | 16 | 7.53×10^-4^ | 2.09×10^-5^ | -0.860 | 0.394 |
|  |  | CT/CC | 31 | 7.58×10^-4^ | 1.98×10^-5^ |  |  |
|  | RD_(Cluster 2)_ | TT | 16 | 5.30×10^-4^ | 2.27×10^-5^ | -0.837 | 0.407 |
|  |  | CT/CC | 31 | 5.36×10^-4^ | 2.52×10^-5^ |  |  |
| rs6985606 | MD_(Cluster 1)_ | TT/CT | 30 | 7.54×10^-4^ | 1.99×10^-5^ | -1.141 | 0.260 |
|  |  | CC | 17 | 7.61×10^-4^ | 2.04×10^-5^ |  |  |
|  | RD_(Cluster 2)_ | TT/CT | 30 | 5.30×10^-4^ | 2.22×10^-5^ | -1.575 | 0.122 |
|  |  | CC | 17 | 5.41×10^-4^ | 2.67×10^-5^ |  |  |
| rs4680 | MD_(Cluster 1)_ | AA/GA | 29 | 7.53×10^-4^ | 1.90×10^-5^ | -1.562 | 0.125 |
|  |  | GG | 18 | 7.62×10^-4^ | 2.11×10^-5^ |  |  |
|  | RD_(Cluster 2)_ | AA/GA | 29 | 5.29×10^-4^ | 2.43×10^-5^ | -1.787 | 0.081 |
|  |  | GG | 18 | 5.42×10^-4^ | 2.28×10^-5^ |  |  |
| rs737866 | MD_(Cluster 1)_ | TT | 29 | 7.59×10^-4^ | 2.14×10^-5^ | 1.227 | 0.226 |
|  |  | CT/CC | 18 | 7.52×10^-4^ | 1.75×10^-5^ |  |  |
|  | RD_(Cluster 2)_ | TT | 29 | 5.36×10^-4^ | 2.69×10^-5^ | 0.883 | 0.382 |
|  |  | CT/CC | 18 | 5.30×10^-4^ | 1.94×10^-5^ |  |  |
| rs1045642 | MD_(Cluster 1)_ | AA/GA | 29 | 7.57×10^-4^ | 1.92×10^-5^ | 0.234 | 0.816 |
|  |  | GG | 18 | 7.55×10^-4^ | 2.21×10^-5^ |  |  |
|  | RD_(Cluster 2)_ | AA/GA | 29 | 5.33×10^-4^ | 2.39×10^-5^ | -0.224 | 0.823 |
|  |  | GG | 18 | 5.35×10^-4^ | 2.56×10^-5^ |  |  |
| rs1128503 | MD_(Cluster 1)_ | AA | 19 | 7.54×10^-4^ | 2.23×10^-5^ | -0.617 | 0.541 |
|  |  | GA/GG | 28 | 7.58×10^-4^ | 1.88×10^-5^ |  |  |
|  | RD_(Cluster 2)_ | AA | 19 | 5.32×10^-4^ | 2.64×10^-5^ | -0.331 | 0.742 |
|  |  | GA/GG | 28 | 5.35×10^-4^ | 2.32×10^-5^ |  |  |
| rs806368 | MD_(Cluster 1)_ | TT | 15 | 7.55×10^-4^ | 1.59×10^-5^ | 1.561 | 0.103 |
|  |  | CT/CC | 32 | 7.52×10^-4^ | 2.09×10^-5^ |  |  |
|  | RD_(Cluster 2)_ | TT | 15 | 5.34×10^-4^ | 1.83×10^-5^ | 1.102 | 0.247 |
|  |  | CT/CC | 32 | 5.29×10^-4^ | 2.54×10^-5^ |  |  |
| rs2246709 | MD_(Cluster 1)_ | AA | 16 | 7.51×10^-4^ | 1.87×10^-5^ | -1.285 | 0.205 |
|  |  | GA/GG | 31 | 7.59×10^-4^ | 2.06×10^-5^ |  |  |
|  | RD_(Cluster 2)_ | AA | 16 | 5.27×10^-4^ | 2.52×10^-5^ | -1.385 | 0.173 |
|  |  | GA/GG | 31 | 5.37×10^-4^ | 2.35×10^-5^ |  |  |
| rs3192723 | MD_(Cluster 1)_ | TT/CT | 16 | 7.55×10^-4^ | 1.55×10^-5^ | -0.188 | 0.852 |
|  |  | CC | 31 | 7.57×10^-4^ | 2.24×10^-5^ |  |  |
|  | RD_(Cluster 2)_ | TT/CT | 16 | 5.32×10^-4^ | 1.85×10^-5^ | -0.317 | 0.753 |
|  |  | CC | 31 | 5.35×10^-4^ | 2.70×10^-5^ |  |  |
| rs2239622 | MD_(Cluster 1)_ | AA/GA | 37 | 7.58×10^-4^ | 2.18×10^-5^ | 1.012 | 0.317 |
|  |  | GG | 10 | 7.51×10^-4^ | 1.08×10^-5^ |  |  |
|  | RD_(Cluster 2)_ | AA/GA | 37 | 5.35×10^-4^ | 2.69×10^-5^ | 0.554 | 0.583 |
|  |  | GG | 10 | 5.30×10^-4^ | 1.06×10^-5^ |  |  |
| rs2565055 | MD_(Cluster 1)_ | TT/GT | 22 | 7.50×10^-4^ | 1.33×10^-5^ | 0.471 | 0.832 |
|  |  | GG | 25 | 7.52×10^-4^ | 2.34×10^-5^ |  |  |
|  | RD_(Cluster 2)_ | TT/GT | 22 | 5.26×10^-4^ | 1.65×10^-5^ | 0.834 | 0.213 |
|  |  | GG | 25 | 5.31×10^-4^ | 2.80×10^-5^ |  |  |
| rs6265 | MD_(Cluster 1)_ | TT | 12 | 7.59×10^-4^ | 1.98×10^-5^ | 0.635 | 0.529 |
|  |  | CT/CC | 35 | 7.55×10^-4^ | 2.04×10^-5^ |  |  |
|  | RD_(Cluster 2)_ | TT | 12 | 5.39×10^-4^ | 1.84×10^-5^ | 0.873 | 0.387 |
|  |  | CT/CC | 35 | 5.32×10^-4^ | 2.60×10^-5^ |  |  |

^*^p<0.05. DTI, diffusion tensor imaging; MD, mean diffusivity; MMT, methadone maintenance treatment; RD, radial diffusivity; SD, standard deviation; SNP, single nucleotide polymorphism.
